# The Coupled Straintronic-Photothermic Effect

**DOI:** 10.1038/s41598-017-18411-w

**Published:** 2018-01-08

**Authors:** Vahid Rahneshin, Dominika A. Ziolkowska, Arthur McClelland, Jaya Cromwell, Jacek B. Jasinski, Balaji Panchapakesan

**Affiliations:** 1 Small Systems Laboratory, Department of Mechanical Engineering, Worcester Polytechnic Institute, Worcester, MA 01609 USA; 2 Conn Center for Renewable Energy Research, University of Louisville, Louisville, KY 40292 USA; 3 Faculty of Physics, University of Warsaw Pasteura 5, 02-093 Warsaw, Poland; 4 Center for Nanoscale Systems, Harvard University, Cambridge, MA 02138 USA

## Abstract

We describe the coupled straintronic-photothermic effect where coupling between bandgap of the 2D layered semiconductor under localized strains, optical absorption and the photo-thermal effect results in a large chromatic mechanical response in TMD-nanocomposites. Under the irradiation of visible light (405 nm to 808 nm), such locally strained atomic thin films based on 2H-MoS_2_ embedded in an elastomer such as poly (dimethyl) siloxane matrix exhibited a large amplitude of photo-thermal actuation compared to their unstrained counterparts. Moreover, the locally strain engineered nanocomposites showed tunable mechanical response giving rise to higher mechanical stress at lower photon energies. Scanning photoluminescence spectroscopy revealed a change in bandgap of 30 meV between regions encompassing highly strained compared to the unstrained few layers. For 1.6% change in the bandgap, the macroscopic photo-thermal response increased by a factor of two. Millimeter scale bending actuators based on the locally strained 2H-MoS_2_ resulted in significantly enhanced photo-thermal actuation displacements compared to their unstrained counterparts at lower photon energies and operated up to 30 Hz. Almost 1 mN photo-activated force was obtained at 50 mW and provided long-term stability. This study demonstrates a new mechanism in TMD-nanocomposites that would be useful for developing broad range of transducers.

## Introduction

Photo-thermal actuation, i.e., the conversion of light into thermal and mechanical work is of significant importance for energy conversion/reconfigurable technologies. Advantages of such photo-thermal mechanisms for transducers include remote energy transfer, remote controllability, control of actuation using number of photons (intensity) and photon energies (wavelength), fast actuation (milliseconds), low signal to noise ratio, high stored elastic strain energy densities with hyperelastic elastomers and scalability at different length scales using batch fabrication and high-volume semiconductor manufacturing. However, only few materials exist that can convert light into mechanical work. Azobenzene liquid crystal elastomers were one of the first materials to exhibit photomechanical effect^1^. However, their application required two different light sources for reversible thermal switching (420 nm and 365 nm) between an extended *trans* and a shorter *cis* configuration^2^.

Recently, various nanomaterials including nanoparticles, nanowires, carbon nanotubes, and graphene were mixed into different polymers from hyperelastic rubbers^3,4^, shape memory polymers^5^ and liquid crystal elastomers^6–8^ and triggered using near infra-red light for photo-thermal actuation. In hyperelastic elastomers, based on the pre-strains, the samples when heated, can either expand, exhibit zero stress or contract in volume and can enable high photo-thermal strain energy densities^3,4^. Large pre-strains result in large volume contraction on photo-thermal heating due to entropic elasticity. The mechanism of photo-thermal actuation in these nanocomposites is the non-radiative decay of photons resulting in localized thermal effect. While, applications of photo-thermal actuators based on nanomaterials inside thermo-responsive polymers are growing rapidly^6,7,9–14^, there is still a need for material design that is simple, enables tunable optical absorption, and reversible photo-thermal response. Two-dimensional (2D) nanomaterials based on Transition Metal Dichalcogenides (TMDs) encompass large optical absorption (10^7^ m^−1^)^14,15^ and strain engineering of such TMDs incorporated into hyperelastic matrix presents new opportunities in photo-thermal transducers which we have explored in this paper.

Strain engineering, i.e., the change in electronic properties using mechanical deformation is an important concept in condensed matter physics and material science that is being exploited in 2D materials due to their ultra-large mechanical strength (capable of withstanding 10–20% strains) and possessing a bandgap^16–21^. Strain engineering in 2D layered materials brings potential benefits such as those explored in graphene including; strain induced bandgap opening^22^, strain enhanced electron-phonon coupling^23,24^, non-uniform strain induced pseudo-magnetic field^25^ and even strain engineered self-assembly of hydrogen on graphene surfaces^26^ In TMDs strain engineering has brought benefits such as bandgap engineering of single and bi-layers^20^, exciton funneling effects^21^, and selective chemical adsorption of gas molecules on strained surfaces^27^.

Compared to graphene, which is just a layer of carbon atoms and has no bandgap, TMDs are stacks of triple layers with transition metal layer between two chalcogen layers and they also possess an intrinsic bandgap. The elastic modulus and the breaking strength of an ideal defect-free single-layer molybdenum disulfide (MoS_2_) is expected to be E^2D^/9, where E^2D^ is the in-plane stiffness as per the theory of rupture and flow in solids^28,29^. The in-plane stiffness of monolayer MoS_2_ is reported as 180 ± 60 Nm^−1^ corresponding to an effective Young’s modulus of 270 ± 100 GPa, which is comparable to that of steel^28^. Breaking occurs at an effective strain between 6 and 11% with the average breaking strength of 23 GPa^28^.

The large breaking strength makes strain engineering an interesting prospect in TMDs, as the bandgaps in TMDs are highly sensitive to strains. Bulk form of MoS_2_ has an indirect band gap of ~1.29 eV^30^ and strain engineering has no effect on such bulk materials and does not produce any significant chromatic mechanical response. On the other hand, single layer MoS_2_ has a band gap of 1.8–1.9 eV^30^; bi-layer has a bandgap of 1.5–1.6 eV^20^ and strain engineering can modulate electronic and optoelectronic properties. With decreasing thickness, the past experiments have revealed a progressive confinement-induced shift in the indirect gap from the bulk value of 1.29 eV up to 1.90 eV^30^. The in-plane structure of MoS_2_ is determined by strong covalent bonds resulting from the overlap between the *4d* and *3p* electron orbitals of Mo and S, respectively^28^. In few layer 2H-MoS_2_, the bandgap goes from direct-to-indirect at >1% strains^20^ and undergoes semiconductor-to-metal transition at 10–15% strains^31^.

Several techniques are realized for strain engineering of 2D layered materials including bending/elongating a flexible substrate^20^, piezoelectric compression^32^, exploiting thermal expansion mismatch^33^, creating artificial atoms through indentation/capillary forces^34^, and controlled wrinkling^21^. However, large area photo-thermal devices based on these mentioned techniques of strain engineering of 2D layered TMDs are yet to be explored with hyperelastic materials.

Utilizing exfoliated nanoparticle MoS_2_ suspensions, filtration and layer-by-layer process of nanocomposite fabrication, and strain transfer from polymeric substrate, here we present the first direct evidence of chromatic optical absorption and reduction in bandgap due to localized strains in TMDs directly affecting the photo-thermal response of a nanocomposite at the macroscopic length scales. Our work is also the first locally strain engineered 2D layered device to perform mechanical work using this coupled straintronic-photo-thermic effect.

## Results

**Figure 1:**
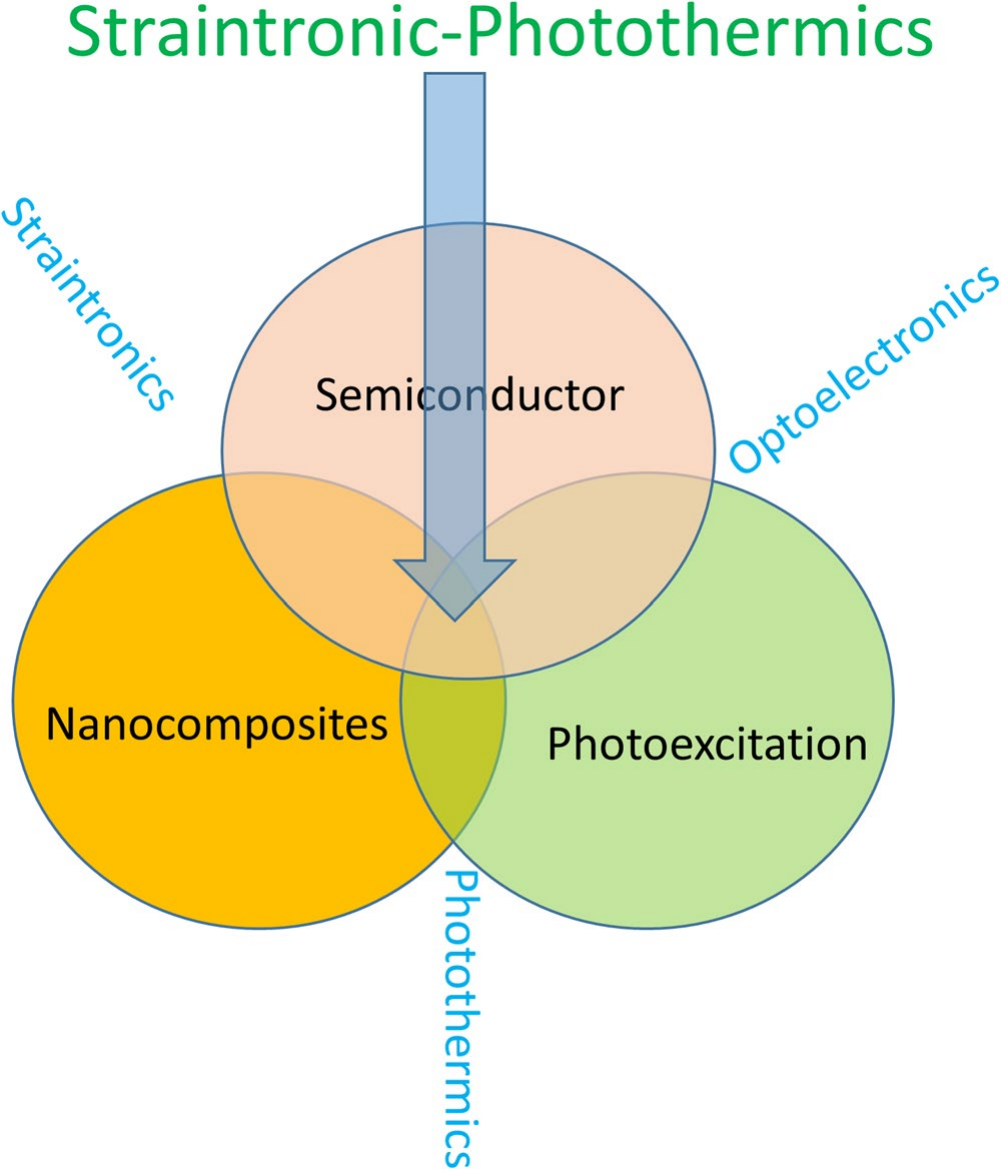
The Straintronic-Photothermic Effect: This effect is the coupling of optoelectronics, straintronics, and photothermics in 2D layered TMD based nanocomposites.

Figure 1 describes the straintronic-photothermal effect. This effect arises due to coupling of electronic properties of semiconductors, thermal properties of polymers and photoexcitation. The mechanism is the coupling between localized strains, change in the bandgap and optical absorption of 2D TMDs transducing into a thermal effect that expands/contracts the hyperelastic molecular chains. This effect is chromatic (depends on the wavelength of light), fully reversible, bandgap dependent and produces elastic mechanical response. This effect only happens in layered semiconductors and not in bulk materials. The strain induced lowering of the bandgap in 2D layered semiconductor is coupled to the enhanced optical absorption. The thermal energy generated during the process of photoexcitation across the bandgap is coupled to the hyperelastic molecular chains that make them highly mobile internally. In zero strain and strained semiconductor nanocomposites, we observed chromatic mechanical response.

**Figure 2:**
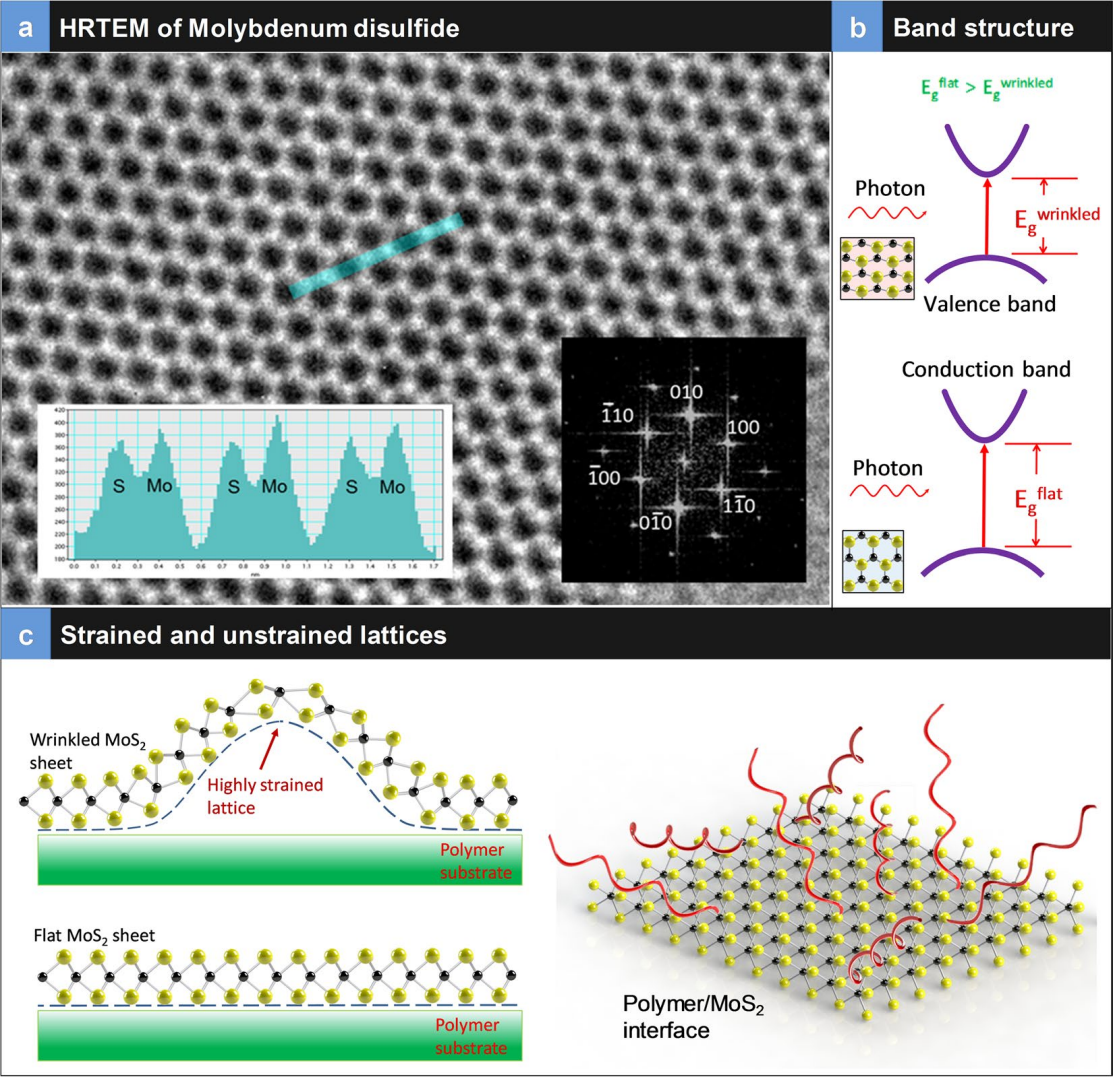
Structure of TMDs: (**a**) High-Resolution Transmission Electron Microscopy (HRTEM) of single layer TMD. Insert shows the Fourier Transform and the intensity profile measured along the blue line shown in the HRTEM image, The atomic positions of S and Mo atoms is indicated; (**b**) schematic of the band structure of flat and wrinkled or wrinkle suggesting reduction in bandgap energy with strain; (**c**) schematic of the highly strained and flat MoS_2_ atomic sheets on polymer substrate; the interface suggests that molecular chains are entangled on the sheet that aids in effective heat transfer from the additive into the polymer.

The High-Resolution Transmission Electron Micrograph (HRTEM) of the MoS_2_ structure is presented in Fig. 2(a) with atoms of S and Mo indicated. The distances a_1_, a_2_ and a_3_ measured from this image are close to the 2.8 Å value of the Mo–Mo interatomic distances in 2H-MoS_2_^35^. The insert in Fig. 2(a) presents the Fast Fourier Transform (FFT) of the HRTEM image indexing the crystallographic planes of the single layer 2H-MoS_2_. Small distortions and apparent non-uniform intensity distribution of the spots in FFT are due to a slight tilt of the flake normal to the electron beam. In the 2H-MoS_2_ lattice, each Mo atom is located at the center of a trigonal prism created by six S atoms. The lattice constant of 2H-MoS_2_ was reported to be: *a* = *b* = 3.14 Å^36^ and *c* = 12.3 Å^37^. In the bulk form of MoS_2_ with an indirect band gap of ~1.29 eV^30^, the photon absorption process is dominated by electronic polarization, and as a result, no wavelength selective optical absorption is observed since no significant electronic transition happens from the valence to the conduction band. However, as the number of MoS_2_ layers decreases, the indirect band gap becomes large and the material changes into a direct band gap semiconductor with E_g_ ~1.9 eV in a single layer^30^ with intrinsic photoluminescence^38^. Figure 2(b) presents the schematic of the band structure of flat versus locally strained architecture for 2H-MoS_2_. Inserts next to the band structures show the flat and strained honeycomb structure of the semiconducting phase of 2H-MoS_2_. Figure 2(c) presents the schematic of the strained versus flat actuators. Local strain engineering creates wrinkles. The schematic of a flat sheet with polymer chains in contact is also presented in Fig. 2(d). Since these are layered structures that is transferred from the filter membrane to the polymer, it is expected that the contact between the MoS_2_ and polymer is primary adhesion and the molecular chains are interacting with MoS_2_ layers entropically. This contact enables the thermal energy be transferred from the MoS_2_ additive to the polymer chains enabling a photo-thermal response due to entropic elasticity.

**Figure 3:**
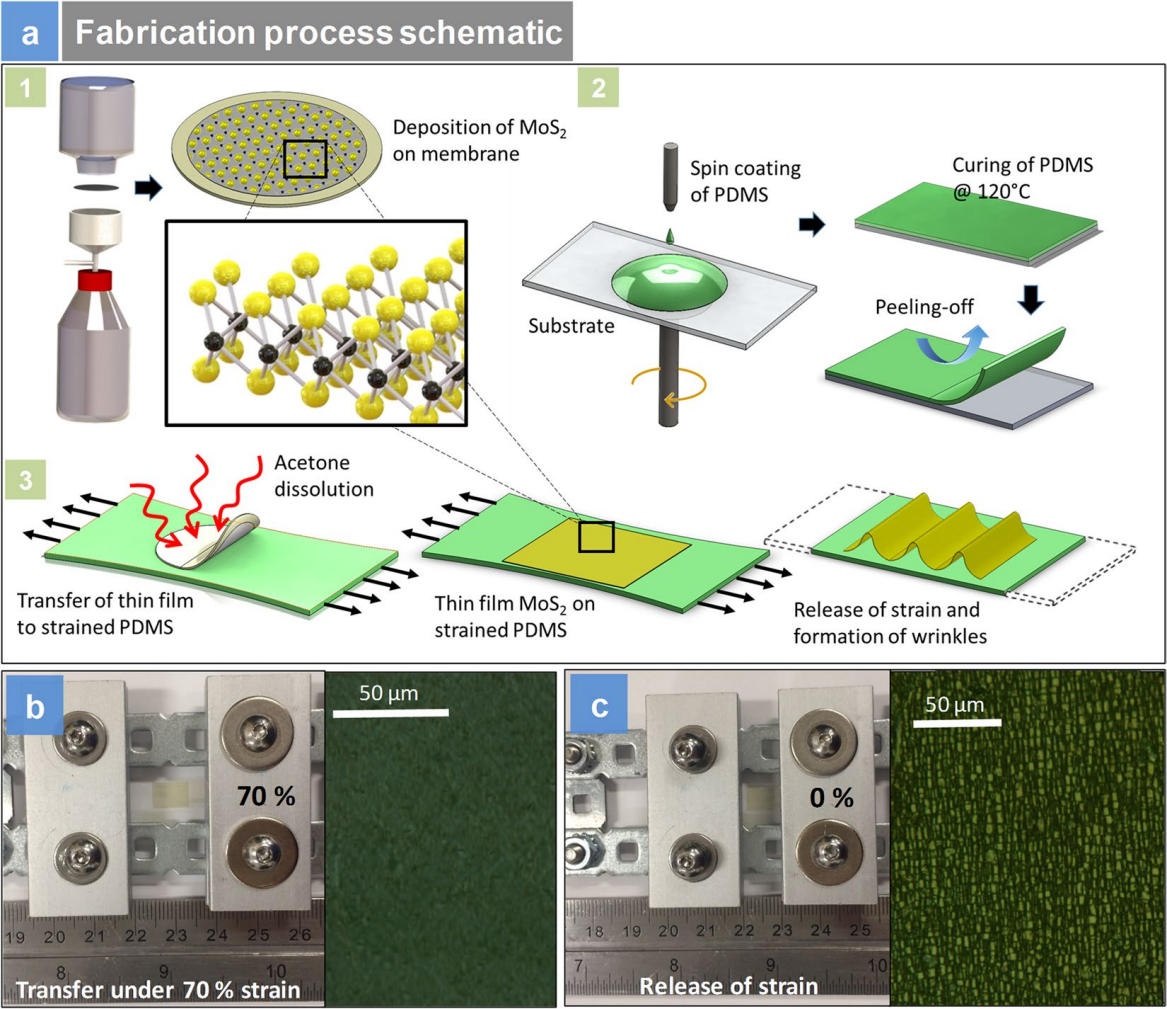
Device Fabrication: (**a**) Schematic of the fabrication of the actuator and release of strains and formation of wrinkles; (**b**) gadget used for strain transfer at 70% strains and optical image of the flat sample before the strain is released; (**c**) gadget at 0% strain and corresponding optical image after the strain is released.

Figure 3 shows the fabrication schematic, strain transfer and optical images of the samples in Polydimethylsiloxane (PDMS). The starting point of the photo-thermal actuators is the filtration of commercially available 2H-MoS_2_ suspensions to form a film^14^. Initial characterization revealed few layered MoS_2_ nanoparticle suspensions had a particle size of ~100 nm. Atomic force microscopy (AFM) measurements suggested from 1–6 layers^14^ and were used for preparing the photomechanical actuators. The suspensions were filtered through a membrane using simple filtration (no vacuum is applied). Filtration resulted in optically smooth layers on top of the filter membrane of a specific thickness. A layer of PDMS is then spin coated onto a glass slide, cured and peeled off. The peeled off layer of PDMS is then set up between clamps and strain of 70%, is applied to the PDMS layer by stretching the sample. The filter membrane consisting of 35 nm or 70 nm 2H-MoS_2_ thin film is transferred to the strained layer, and the membrane is dissolved in acetone, leaving behind the thin film of 2H-MoS_2_ on the strained PDMS layer. We made two different thickness of film namely 35 nm and 70 nm film to assess the straintronic-photothermal response. Releasing the strains suddenly results in the formation of wrinkles. Figure 3(b) and (c) presents the clamps with PDMS thin film before, and after releasing the strain, respectively. Once the strain is released as presented in Fig. 3(c), lamellar structures are seen under an optical microscope suggesting the appearance of the wrinkles.

**Figure 4:**
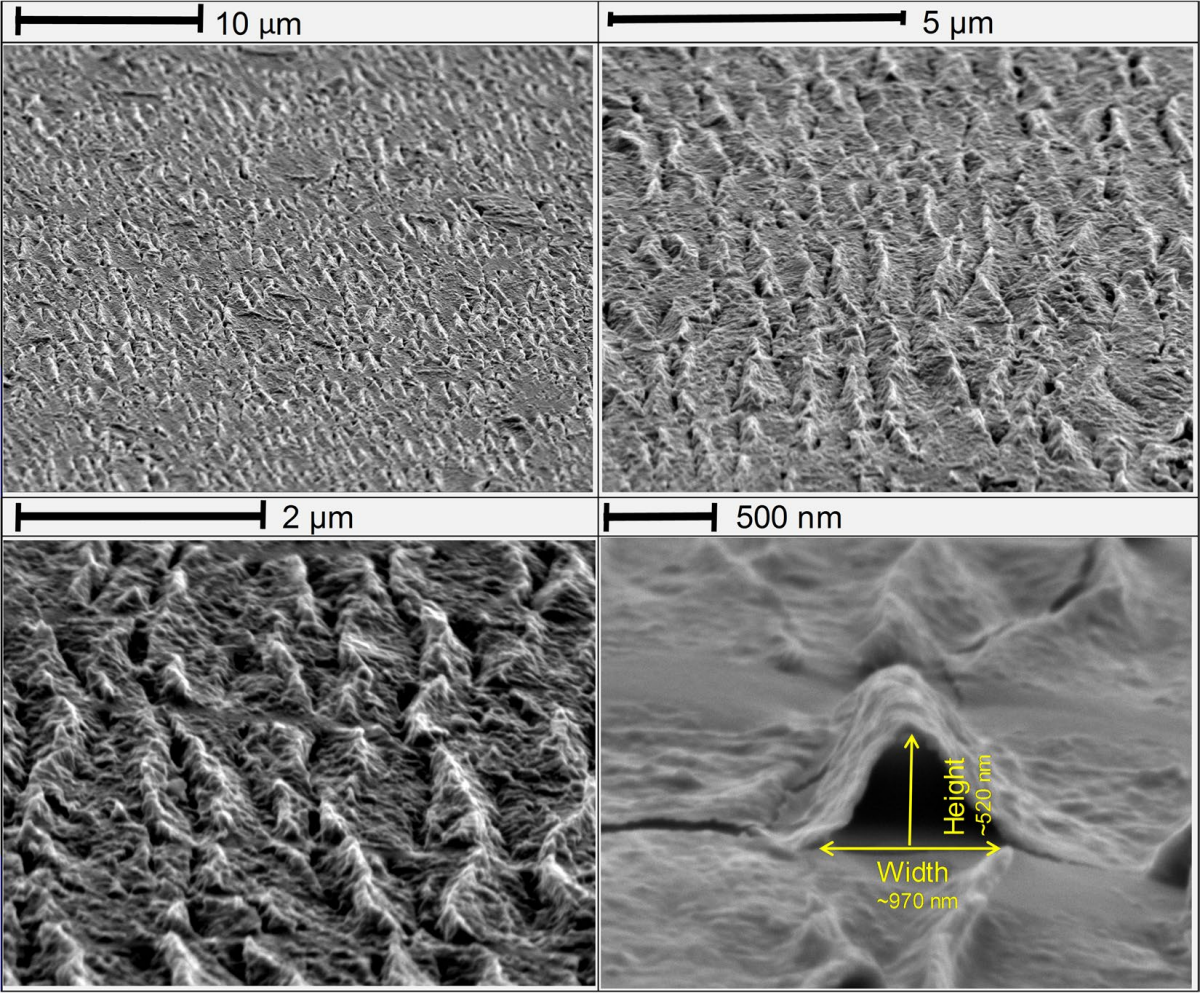
Scanning Electron Microscopy of Wrinkles: High angle SEM image of thousands of wrinkles at different magnification. (**a**–**c**) Highly reproducible wrinkles at large scale; (**d**) A single wrinkle seen between flat layers in the 500 nm image. The image suggests these are highly reproducible structures. A single wrinkle is almost 1 µm in width and 520 nm in height.

Figure 4(a–d) presents the scanning electron micrograph (SEM) of the wrinkled architecture at different magnification. Strain transfer from an elastomeric substrate such as PDMS resulted in thousands of wrinkles at a large scale. Here we have used the same strain transfer from a polymer substrate at a large scale. Figure 4(d) presents the SEM image of a single wrinkle between flat layers, measuring 520 nm in height and 970 nm in width. Figure S_2_ (Suppl.) presents the AFM image of the wrinkles on the polymer substrate. The lamellar structure is seen from the top extending to several microns. The height image from the AFM is presented for different regions showing ~70–80 nm consistently in height. The height and width of over 100 wrinkles are listed in Table S_1_ (Suppl.), reporting average values of 87 nm and 553 nm for height and width of wrinkles in 35 nm film. Castellanos-Gomez *et al*. showed such wrinkles are generated from a single sheet of MoS_2_ consisting of 3–4 layers using the strain transfer from polymer for studying bandgap engineering in mechanically exfoliated samples^21^. We have used this process to transfer strains into nanoparticles of MoS_2_ formed into a thin film. The films, therefore, are not one continuous sheet, but quasi-continuous sheets consisting of few layer nanoparticles ~100 nm in diameter each. Such films also create stable wrinkles without disintegrating into the substrate. Releasing the strain from the elastomeric substrate results in delamination induced buckling, of our quasi-continuous sheets^39^. We were able to produce wrinkles that were 12–170 nm in height and 300 to 700 nm in width. Previous work has shown that, the maximum uniaxial tensile strain ε accumulated on top of the winkles is estimated as^39^:

1 $${\rm{\varepsilon }} \sim {{\rm{\pi }}}^{2}{\rm{h}}\,{\rm{\delta }}/(1-{{\rm{\sigma }}}^{2}){\lambda }^{2},$$

where σ is the Poisson’s ratio of MoS_2_ (0.125), h is the thickness of the flake and δ and λ are the height and width of the wrinkle respectively. However, this model assumes the sheet to be continuous and the strains are often overestimated for thin films of nanoparticles. A modification of this model is thus necessary to accurately predict the strain profile in our quasi-continuous sheets. The analytical strain model for van der Waals packed films was developed based on the original work by Vella *et al*.^39^. The new strain relationship for the nanoparticle films is thus given as:

2 $${\varepsilon }_{x}(x,z)\cong -\frac{2{\pi }^{2}z\delta }{{\lambda }^{2}}cos\frac{2\pi x}{\lambda }$$

where, *x* is the horizontal distance from apex of the wrinkle and *z* the distance from the mid-plane of each individual nanoparticle. The details of this model can be found in the supplementary document and Figure S_1_.

We calculated strains from >100 different wrinkles based on the height, width and thickness measurements as per equation 2. Supplementary Tables S_1_–S_4_ presents the statistical information on the width, height and length of each wrinkle measured from the AFM images. The tabulated results show anywhere between 0.2–1.3% strains were achieved in the wrinkles. For the 35 nm film (Table S_1_), average strains from >100 different places suggested a 0.6% and for 70 nm film (Table S_3_), strains of 0.3% calculated using equation 2.

**Figure 5:**
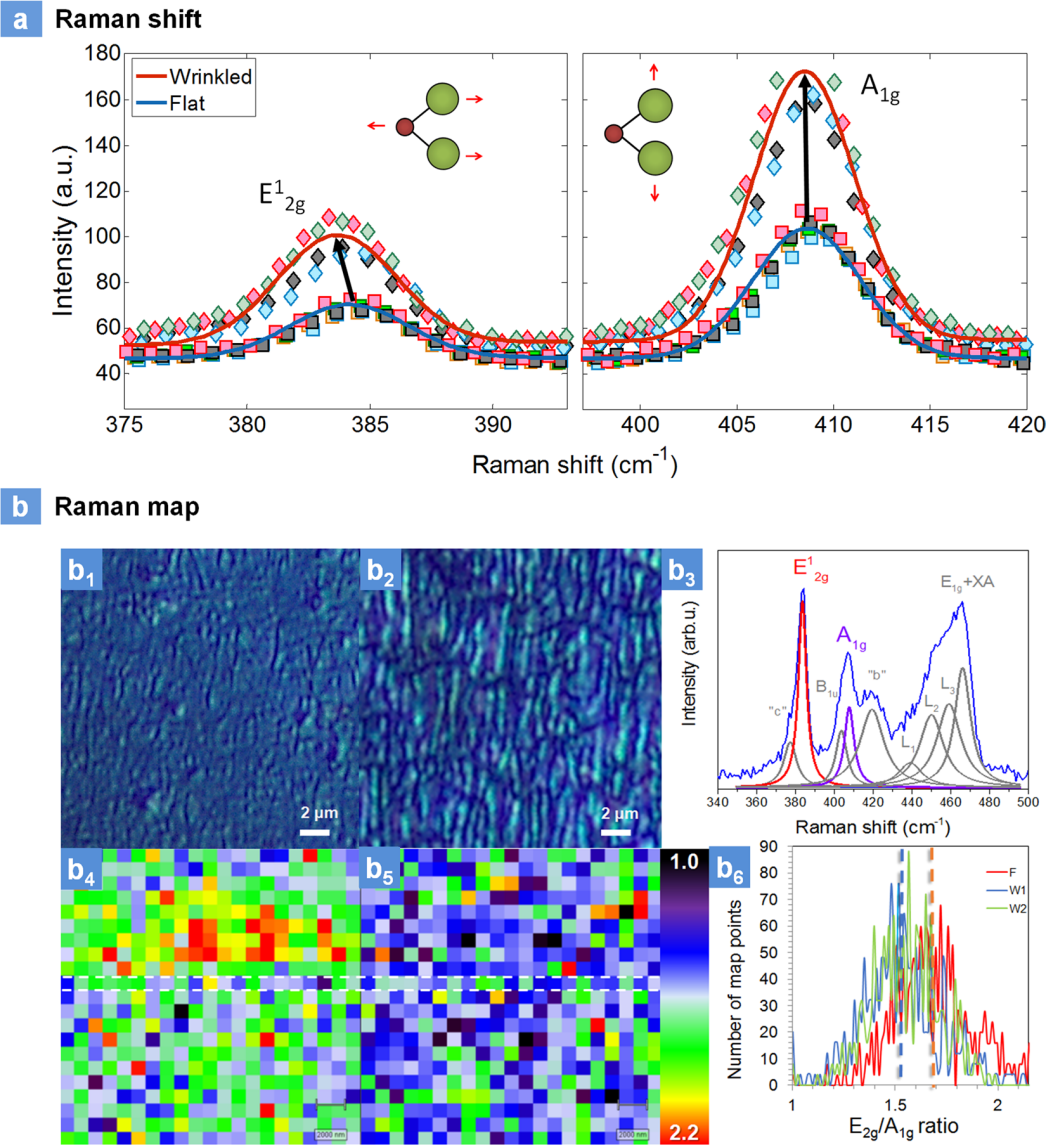
Raman Spectroscopy: (**a**) Raman shift between flat and wrinkled wrinkle architectures (excitation source: 532 nm); (**b**) Optical images of flat (b_1_) and wrinkled (b_2_) samples showing a clear difference of the strained surface; (b_3_) resonant Raman scattering spectrum (excitation source: 633 nm) of the wrinkled samples showing a complex vibrational mode structure; (b_4_–b_5_) Raman maps of E_2g_/A_1g_ intensity ratio recorded from 400 µm^2^ areas of flat and wrinkled samples and (b_6_) histograms of E_2g_/A_1g_ intensity ratio values measured from three Raman maps (F-flat/unstrained, W-wrinkled/strained). Each histogram represents 441-pixel points.

Figure 5 presents the Raman spectroscopic measurements between flat and wrinkled actuator samples. Raman spectroscopy is a powerful tool to characterize 2D nanomaterials such as graphene and MoS_2_ subjected to a uniaxial strain. Initial work by Li *et al*., showed coupling between electronic transitions and phonons are found to become weaker when the layer number of MoS_2_ decreases, attributed to the elongated intralayer atomic bonds in ultrathin MoS_2_^40^. Figure 5(a) presents the Raman measurements between flat and wrinkle samples measured using 532 nm laser wavelength. Clear changes in the E^1^_2g_ and the A_1g_, modes are seen between the flat and wrinkled actuators. The A_1_g mode that corresponds to the sulfur atoms oscillating out-of-plane is less affected than the E^1^_2g_ mode which is the sulfur and molybdenum atoms oscillating parallel to the crystal plane. The slight shift of the A_1_g peak observed on the wrinkled MoS_2,_ and the larger Raman shift of the E^1^_2g_ peak (~0.75 cm^−1^) are in good agreement with recent reports on strain engineered MoS_2_^21^. In the past, in our few layer nanocomposites, applying global macroscopic uniaxial tensile strain of 10% using a custom made strain gadget resulted in the shift of the E_2g_ mode by about −1.7 cm^−1^ and negligible change in A_1g_^14^. However, those experiments were performed by measuring the Raman shift versus intensity while the strain was still held by the gadget. In the localized strain engineered sample as reported here, the Raman shift versus intensity was measured after the strain was removed from the samples. The smaller change in Raman wavenumbers is expected due to relaxation of the samples after removal of the strain resulting in lowering of Raman wave numbers and due to higher thickness of the film compared to single sheet of few layers.

Raman mapping was conducted over large area to understand the shift in E_2g_/A_1g_ between strained and unstrained samples. Resonant Raman scattering studies were performed at room temperature as presented in Fig. 5(b). Figure 5b(b_1_–b_2_) are the optical images of unstrained (flat) and locally strained actuators showing the visible roughness of the strained sample. Figure 5b(b_3_) is the resonant Raman scattering (measured using 633 nm laser) of the strained few layer 2H-MoS_2_ samples. We have studied resonant Raman scattering of MoS_2_ few layers based on number of layers in our past reports^14^. Resonant Raman spectra of the MoS_2_ films, both wrinkled as well as flat, showed a complex vibrational modes structure with the same Raman features as the few-layer MoS_2_. In addition to the first order E^1^_2g_ (~384 cm^−1^) and A^1^_g_ (~409 cm^−1^) peaks, the spectra contained a number of intense second-order peaks located between 360 cm^−1^ and 660 cm^−1^ Raman shifts. This includes the “b” (~420 cm^−1^) peak associated with the emission of a dispersive quasi-acoustic phonon and simultaneously an optical E^2^_1u_ phonon along the c-axis, the “c” (~380 cm^−1^) peak due to the emission of a E^2^_1u_ (Γ) phonon, and the B_1u_ (Γ) (~404 cm^−1^) peak, which together with the A_1g_ (Γ) peak form a Davydov couple. Several significant changes of the spectrum are observed with strains for the few layer nanocomposite. Compared to the flat samples in Fig. 5b(b_4_), and Fig. S_3_ (suppl.), the A_1g_, E_1g_ + XA, and “b” peak intensities are partially increased in the strained samples. Fig. S_3_ (suppl.) shows the Raman mapping of flat and wrinkled sample corresponding to the region represented by white lines in Figure 5(b_4_–b_5_). For example, the “b” peak shows higher intensity for the locally strained sample compared to the unstrained flat counterparts which is an indicative of the phonon involved that has a wave vector along the c-axis. Similarly, the A_1g_ is the out-of-plane vibrational mode is affected by the strain. The non-uniformity between resonant Raman spectra of the strained samples suggests the non-homogeneity of strain in the wrinkled samples.

Raman mapping was performed, and 21 × 21 spectral maps were collected from 20 µm × 20 µm regions of flat and wrinkled samples over 16 hours as presented in Figure 5b(b_4_–b_5_). The mapping of the large area of the sample showed an unambiguous reduction of E_2g_/A_1g_ intensity ratio in wrinkled samples compared to flat actuators (Figure 5b(b_6_)). The histograms of E_2g_/A_1g_ intensity ratio values measured from three Raman maps: one flat, and two wrinkled ones, showed a good statistical evidence of this change. The average shift in E_2g_/A_1g_ intensity ratio is about 0.2 (a change of ~11%). The decrease in E_2g_/A_1g_ intensity ratio is indicative of strain transfer to the additives across a large area of the substrate, suggesting that this process is highly scalable for future device applications.

**Figure 6:**
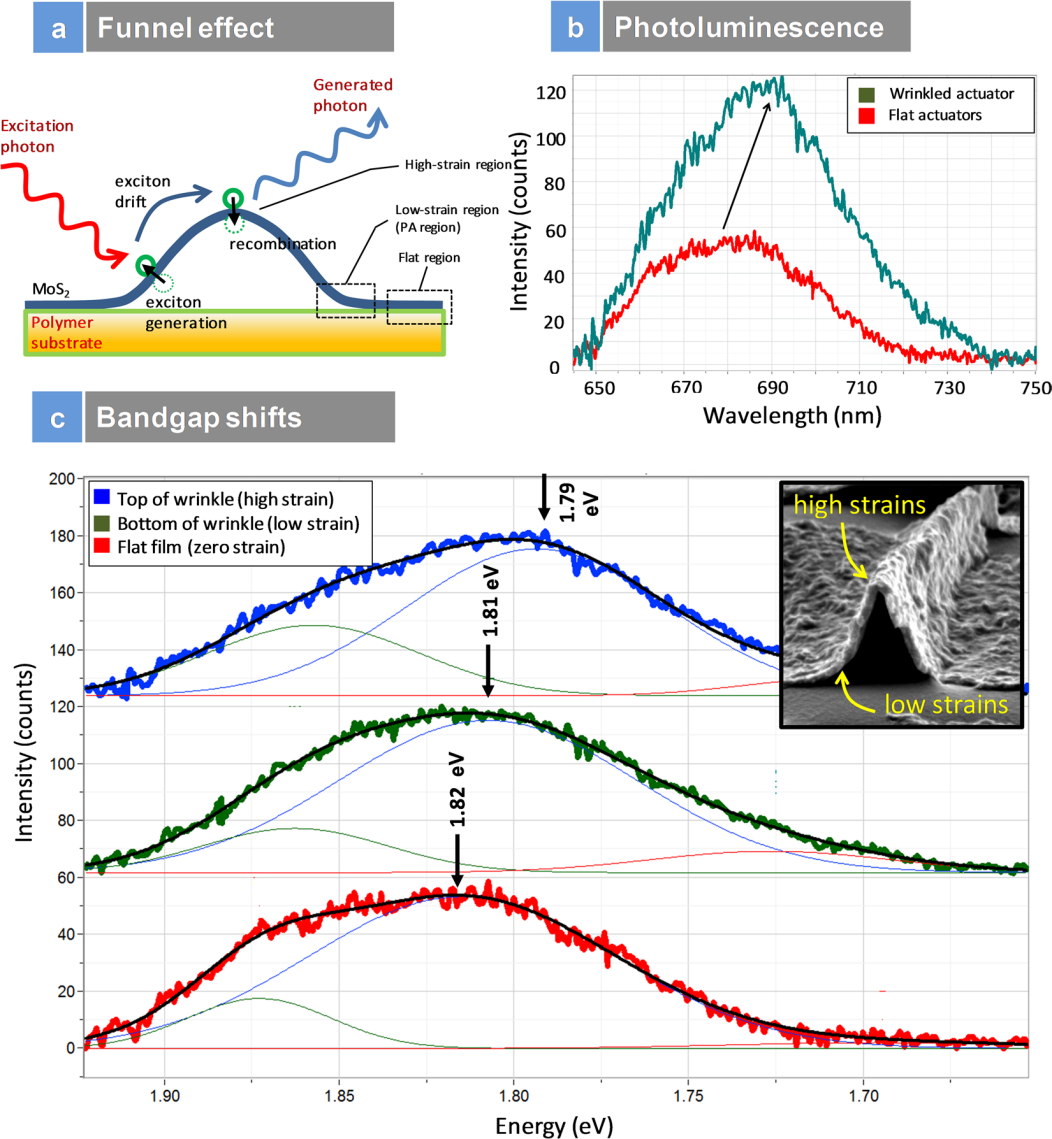
Scanning Photoluminescence Spectroscopy: (**a**) Schematic of funnel effects suggesting exciton drift; (**b**) PL intensity of flat and wrinkle actuators at 35 nm film thickness; (**c**) bandgap shifts with strains; graphs compare top of wrinkle to bottom of wrinkle and flat film; insert is SEM image of the different regions mapped using scanning PL spectroscopy.

Photoluminescence spectroscopy (PL) is a powerful technique to unambiguously show the reduction in bandgap in TMDs with strains. Here, we see that the nanoparticle sheets does exhibit distinct PL spectrum with and without strains. Figure 6(a) presents the schematic of the funnel effects due to exciton drift due to the strains. The top of the wrinkle is the point of the highest strain and the bottom of the wrinkle, where the strain is lowest. Thus, the wrinkle is a geometric structure where strain gradients are established from top to bottom and thus can enable exciton funneling from regions of low strain towards high strain regions^21^. Figure 6(b) presents the photoluminescence spectra of the flat and wrinkled nanoparticle sheets. Both the samples were prepared identically, and the only difference was, application of strain for the sample exhibiting the wrinkle architecture. Since these sheets were 35 nm thick, we expected the photoluminescence to be small compared to single layers, to begin with even for flat sheets. However, they do exhibit distinct photoluminescence as seen in the spectra in Fig. 6(b). The flat sheets had small PL intensity. However, there was an increase in intensity and red shifting of the direct electron bandgap for the wrinkled samples. Bulk MoS_2_ exhibits negligible photoluminescence as there is no direct electron transition and thus, the first thing we can observe is these sheets; they do not act like bulk materials. The photoluminescence intensity was three times stronger for the strained wrinkle architecture for the same thickness suggesting direct electron transition increased due to the application of mechanical deformation. Although few-layer MoS_2_ is an indirect bandgap semiconductor, its photoluminescence spectrum is dominated by the direct gap transitions, at the K point of the Brillouin zone, between the valence band and the conduction band. Here, we see that the A peak is red shifted in the photoluminescence spectra of the flat and the wrinkled samples in line with previous reports on strain engineered few layer MoS_2_. Figure 6(c) presents the bandgap shifts measured using scanning photoluminescence spectroscopy in three regions namely: top of the wrinkle, the bottom of the wrinkle and in a flat film. The insert in this figure presents a wrinkle with regions of low and high strains identified. The strain values for over 100 wrinkles were studied and listed in Table S_1_, reporting strains from 0.2% to 1.3% in our wrinkles. These suggest unambiguously that the bandgap decrease of 30 meV is due to the localized strain engineering and our results agree with the literature reports^21^. The calculated values of local strain based on Equation 2 and the bandgap change in Fig. 6(c) are in good agreement with previous studies; according to the experimental data and tight binding (TB) model presented earlier by Castellanos-Gomez *et al*.^21^ a change of −30 meV in the bandgap (A exciton) corresponds to a strain of ~1.0%. The flat samples showed a bandgap of ~1.82 eV and the strain-induced bandgap was measured as ~1.79 eV, suggesting ~1.6% change in the bandgap due to mechanical deformation. Figure S_4_ (suppl.) presents the PL measurements for both plain PDMS and MoS_2_/PDMS. One can clearly see the A and B peaks in MoS_2_/PDMS sample and not seen in plain PDMS sample.

**Figure 7:**
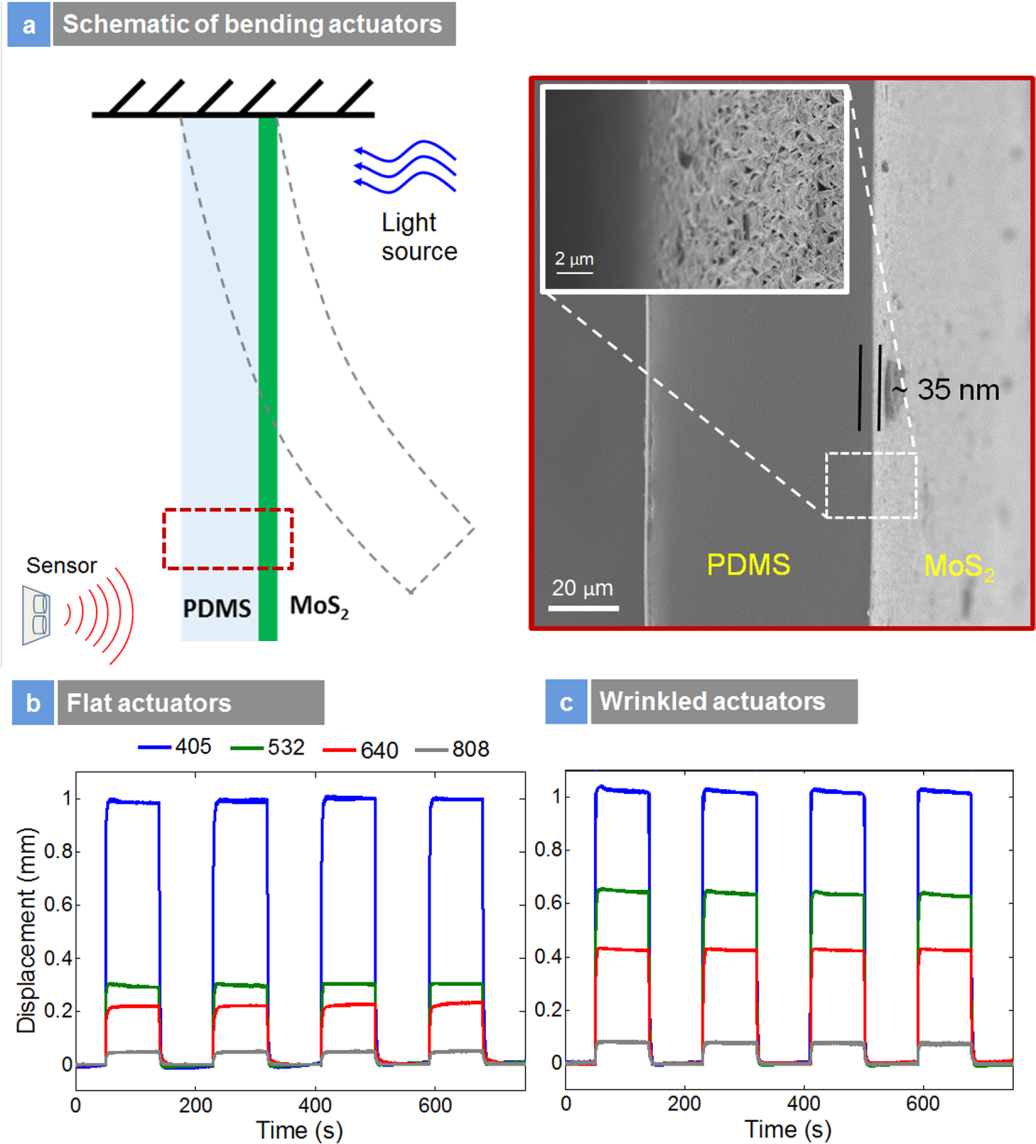
Straintronic Photothermal Actuation: (**a**) schematic of bending actuators and corresponding SEM image; Insert show the wrinkles on polymer substrate; (**b**) photothermal bending of unstrained actuators between 405 nm to 808 nm; (**c**) photo-thermal bending of strained/wrinkle actuators.

Having characterized the actuators with and without strains, we investigated photo-thermal effects of flat (unstrained) and wrinkle (strained) actuators as presented in Fig. 7. Photo-thermal stress measurements were investigated both for flat and wrinkled architectures which are presented in Suppl. S_5_. The power transmitted through the sample at different wavelengths is also presented in Figure S_6_. Both flat and wrinkle based actuators gave rise to chromatic mechanical response, i.e. a differential mechanical response at different wavelengths of light arising due to the optoelectronic transitions across the bandgap. This does not happen in bulk MoS_2_ photomechanical actuators, where the mechanical response is insensitive to the wavelength of light^41^ suggesting that these actuators made from nanoparticle sheets do not act like bulk materials. The chromatic absorption and subsequent mechanical response are first evidence that the semiconductor bandgap is intimately connected to the mechanical response, thus the straintronic-photo thermal effect. This effect also does not happen in thin film semiconductors. For these measurements, no external pre-strains was applied to the sample. At 405 nm, we see that the stress was identical for both unstrained (flat) and strained (wrinkle) architectures. However, for the wrinkle samples, the stress was enhanced at lower photon energies between 532 nm to 808 nm. This enhancement in stress at lower photon energies can only arise from the reduction in bandgap due to strains. The thermal and mechanical effect also follows the reduction in the bandgap. The power transmitted through the samples were higher for the unstrained (Fig. S_6_) samples suggesting more light is absorbed by the highly strained samples at all wavelengths. What is striking is the large values of mechanical stress that can be achieved.

The straintronic-photothermal effect is highly useful for Millimeter scale and Micro-Opto-Mechanical Systems (MOMS). Figure 7 presents the photomechanical response of a millimeter scale bending actuator. Figure 7(a) presents the schematic of the bending actuator. A laser displacement sensor was used to monitor the change in deflection of the cantilever. The actuators consisted of a layer of PDMS and thin layer of MoS_2_. The thickness of the layers was 80 µm for PDMS, and 35 nm for the 2H-MoS_2_ and the length of the actuator was 12 mm. The SEM image presents the cross-section of the PDMS and MoS_2_ layers. The insert in the tilted SEM image presents the thousands of wrinkles. Figure 7(b) presents the displacement of the actuator between 405 nm to 808 nm of the flat nanosheet actuators and Fig. 7(c) presents the displacement of the wrinkled actuators respectively. Remarkably, both the actuators gave rise to the same displacement at 405 nm (energy above the bandgap). Since 405 nm is much larger than the bandgap, this was expected. As we go down the photon energy, the 532 nm, 640 nm and 808 nm excitation resulted in smaller displacement for the flat actuators. The displacements between the 405 nm and 808 nm excitation are almost ten times for the flat actuators. Now coming to the wrinkle actuator sample as presented in Fig. 7(c), we see the same chromatic mechanical response. For the wrinkle architecture, the mechanical response was enhanced at 532 nm, 640 nm, and 808 nm excitations. The mechanical response enhancement was twice at 532 nm, 640 nm, and 808 nm. As the PL spectroscopy suggested a decrease in bandgap of 30 meV, this resulted in improved optical absorption at lower wavelengths resulting in enhanced photo-thermal response. For 1.6% change in the bandgap, the mechanical response enhancement factor is almost two. The results suggest that for lower laser energy input; we get a significantly improved mechanical output compared to the flat actuators. This type of strain induced photo-tunability of mechanical response at lower light energies has not been achieved in other photomechanical materials namely azobenzene liquid crystal elastomers (LCE), carbon nanotubes and nanoparticle based LCEs and this effect opens a new window in the design of photo-thermal systems based on coupled straintronic-photothermic effects. A practical application of this effect would be in tunable photo-thermal nanopositioning^42^.

**Figure 8:**
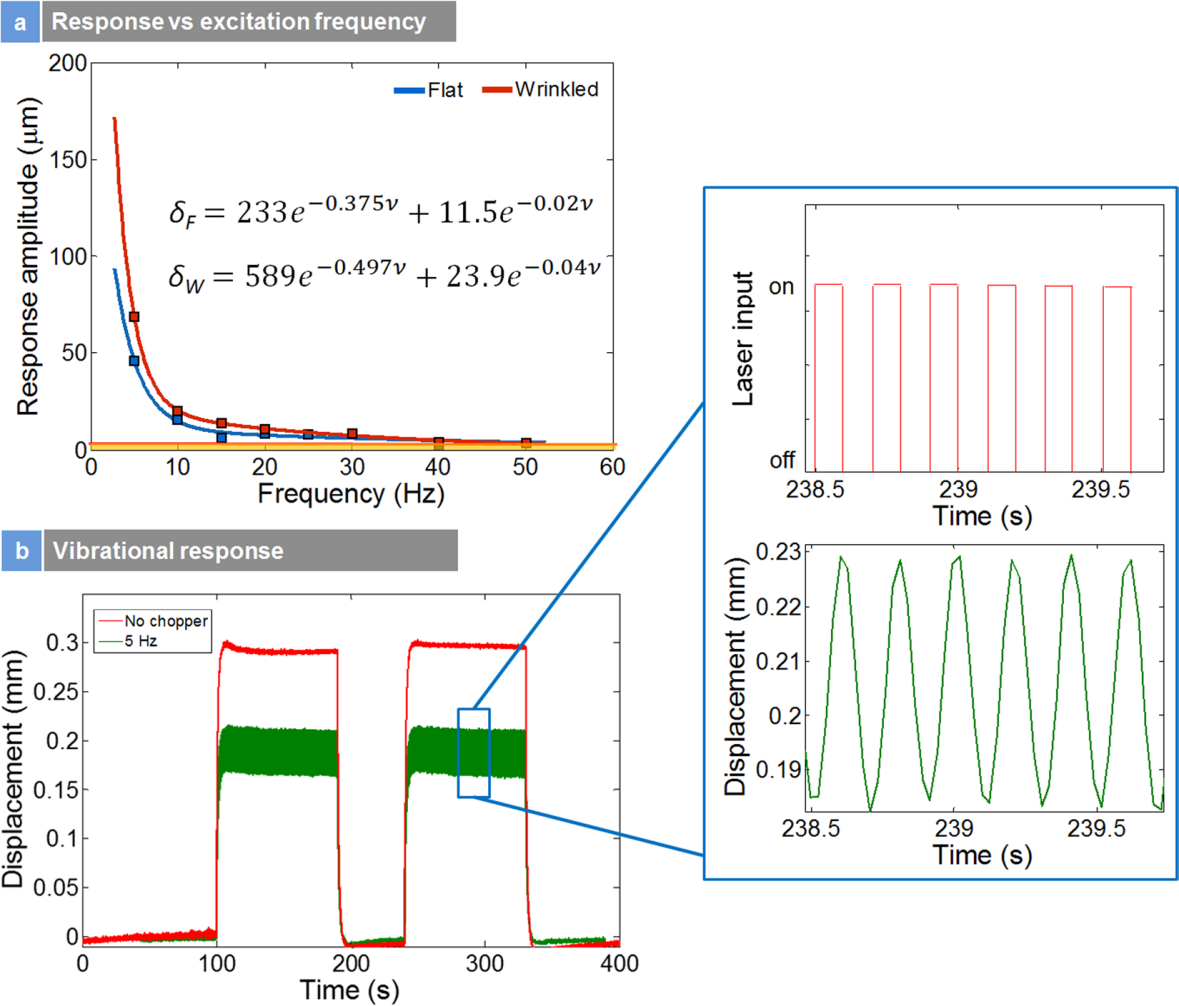
Frequency Response: (**a**) mechanical response versus excitation frequency; (**b**) photothermal vibration response actuation of unstrained actuators at 0 and 5 Hz; Insert is the magnified image of the laser input and mechanical output.

Figure 8 presents the frequency response of the millimeter scale actuators. These actuators were 12 mm in length. The thickness of the PDMS layer was 80 µm. Figure 8(a) presents the mechanical response amplitude versus excitation frequency for both strained and unstrained samples. An exponential decrease was observed in frequency versus mechanical response for both samples. The locally strained samples showed larger response compared to their unstrained flat counterparts at low frequencies. These actuators could be operated up to 30 Hz quite easily (Fig. S_7_ (suppl.)) which is a record by itself for photo-thermal actuators at the millimeter-scale. While these are first demonstrations, with scaling down the actuators to microscopic scales and depending on the thermal mass, one can improve the operability of these actuators to >100 Hz. The relationship between vibrational amplitude (δ) and excitation frequency (v) for both flat and wrinkled actuators is interpolated as:

3 $${{\rm{\delta }}}_{{\rm{F}}}=233{{\rm{e}}}^{-0.375{\rm{v}}}+11.5{{\rm{e}}}^{-0.02{\rm{v}}}$$

4 $${{\rm{\delta }}}_{{\rm{W}}}=589{{\rm{e}}}^{-0.497{\rm{v}}}+23.9{{\rm{e}}}^{-0.04{\rm{v}}}.$$

Figure 8(b) presents the photothermal vibration response of unstrained actuators at 0 and 5 Hz. Insert is the magnified image of the laser input and the mechanical output. The amplitude of the photothermal response is smaller for the 5 Hz actuation. The results also suggest that one can pulse the photo-thermal actuators that would be useful for programmed sensing applications using optical methods.

**Figure 9:**
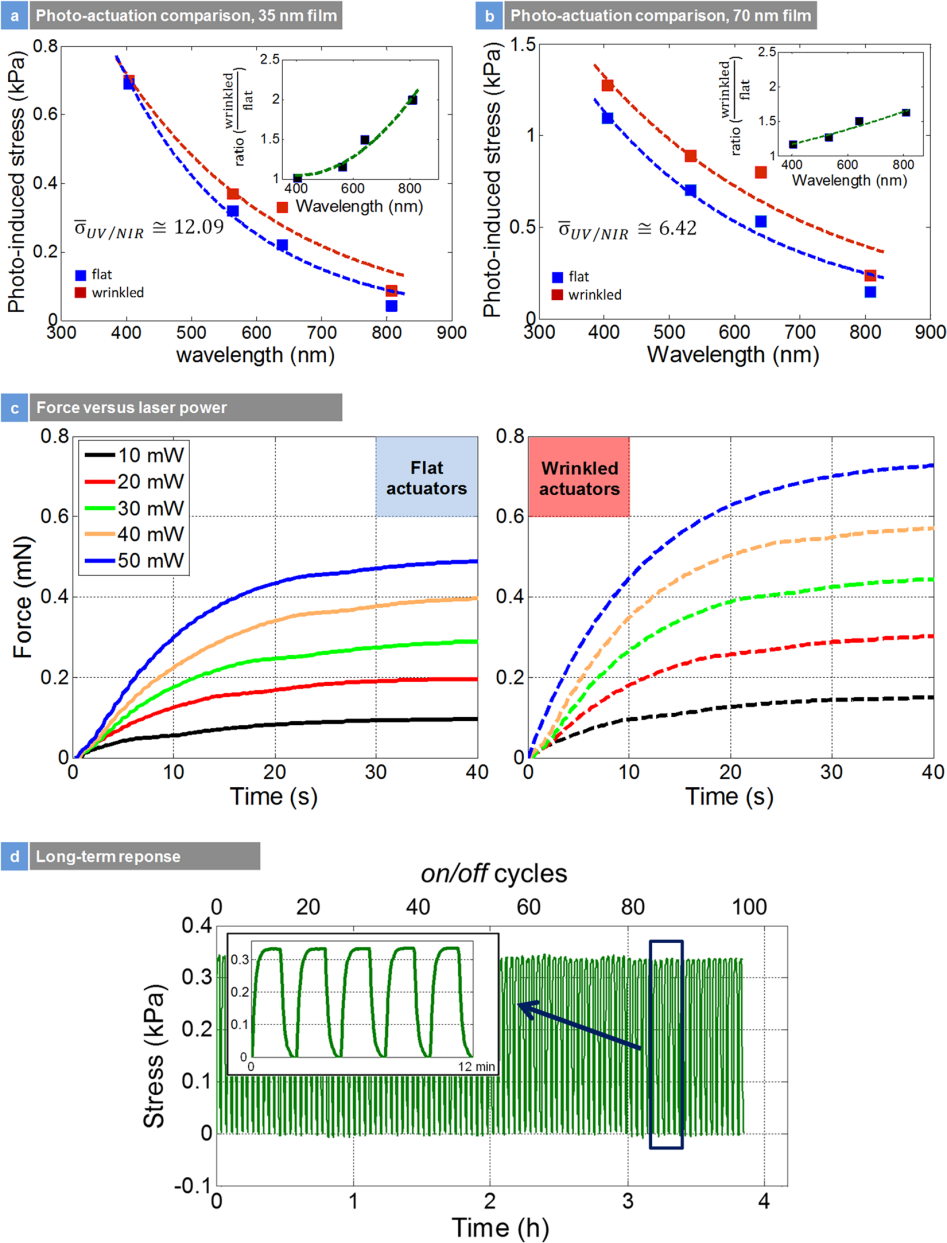
Photoinduced Stress, Force and Long Term Response: (**a**) comparison of photo-activated stress release between unstrained and strained wrinkle macroscopic hyperelastic actuators for 35 nm film; insert is the ratio of the stress between strained and unstrained actuators as a function of photon energy; (**b**) same for 70 nm film; (**c**) force versus laser power comparison for unstrained and locally strained actuators at 640 nm; (**d**) long term response.

Figure 9(a) and (b) are the photomechanical stress response of the flat and wrinkled actuators at various wavelengths. The average ratio of σ_UV/NIR_ (i.e. σ_405/808_) in Fig. 9(a) and (b) is an indication of the amplitude of the chromatic mechanical response. For a 35-nm thick film, a value of 12.09 indicates higher chromatic response compared to 70-nm film with a value of 6.42, also suggesting a higher yield of few layer regions in the 35-nm sample. From these figures, different behaviors of flat versus wrinkle samples are noted; while in 35-nm film, there is a merging point between flat and wrinkle samples at 405 nm, there is not such a point in the 70-nm film. Thus, the thickness plays an important role and the mechanism is gradually changing from layered effect to bulk when increasing the thickness, resulting in disappearance of the merging point.

The insert in Fig. 9(a) and (b) is the ratio of the mechanical stress response of wrinkled versus flat samples. Both the flat and wrinkled architecture has the same pattern of the stress response versus wavelength of light. However, the increase in the ratio of mechanical stress response between wrinkled and flat samples by a factor of two in 35 nm film suggests that the wrinkle architecture samples were unambiguously effective in converting light into thermal energy and mechanical work. We also see that the ratio of the mechanical stress response between wrinkled and flat samples depends on the thickness. For the 35 nm film, a near exponential increase in stress ratio with a decrease in photon energies is seen. However, for the 70 nm film, the increase in stress ratio is approaching a linear behavior. If we plot the ratio of wrinkled to flat actuators with respect to incident wavelength and interpolate the experimental data with the following equation:

5 $$r=a{e}^{bx},$$

The coefficient b for the 35 nm and 70 nm films is 0.00178 and 0.00084, respectively. A decrease in the coefficient with the increase in thickness of thin film is observed. This shows diminishing effect of wrinkling as the thickness of nanoparticle film increases. The exponent coefficient should approach zero for adequately thick film of nanoparticles (>100 nm), where there would not be any improvement in photoactivated stress due to wrinkling. This suggests the thickness limits for the straintronic-photothermal effect is ≤100 nm. It is expected that below 35 nm thickness, this effect will be highly dominant with large amplitudes of stress and exponential mechanical response between UV and NIR wavelengths. Figure 9(c) represents the internal force in different intensity of 640 nm light for both wrinkled and flat samples. Almost 1 mN force was achieved for the wrinkled actuators and they showed higher force compared to flat actuators at all light intensities.

To determine the robustness of this actuator, we did long term experiments in line with previous reports. Figure 9(d) presents the photomechanical stress versus time over 4 hours of continuous operation of the actuators. No drift observed, and we observe that the large-scale wrinkle actuators were stable over long periods. There are conclusions that are deduced from these long-term experiments. First, the photomechanical actuation of the locally strain engineered 2H-MoS_2_ nanocomposite is fully reversible and exhibits a robust actuation mechanism. Second, the photomechanical experiments over long periods of time did not alter the structure of the wrinkles, which would otherwise cause an irreversible change in the amplitude of photomechanical actuation stress (a significant reduction in stress). This also means that the wrinkles are highly stable on the surface of the polymer substrate. Figure S_8_ presents the high angle SEM image of before and after applying cycles of external strains of 60% to a wrinkled sample. The resulting morphology looks the same suggesting no slip, collapse, or permanent changes to the wrinkles. We believe that as long the external strain does not exceed the initial fabrication strains of the wrinkles, they should be stable.

To further understand the mechanism of photo-actuation of MoS_2_/PDMS nanocomposites, iso-strain tests have been done in Dynamic Mechanical Analyzer (DMA-Q800 TA Instruments). A pristine PDMS sample of ~140 µm thick was cut into 8 mm × 30 mm strip and placed in between DMA tension clamps. The sample was stretched to different strain levels. At each of these strain levels, a temperature ramp from room temperature to 60 °C was applied to uniformly heat up the polymer sample and measure the change in the internal force of the sample due to the thermal actuation of PDMS at a fixed length.

In Figure S_9_ (Suppl.), the trend in thermal actuation of pristine PDMS is very similar to photo-actuation of MoS_2_/PDMS nanocomposites. This suggests that MoS_2_ wrinkles act as photo-thermal energy transducing agents that absorb the light energy depending on the bandgap and convert it to thermal energy. The thermal energy is transferred to polymer chains of PDMS, making them expand or contract due to entropic elasticity. Similar to photo-actuation behavior^43^, there are three different regimes in the thermal actuation process: expansion, contraction, and zero-actuation regions. In small pre-strains (0–5%), heating of the sample leads to increase in length, i.e. decrease in the internal stress of the PDMS strip. At medium pre-strains (5–10%), heating does not have a significant effect due to the balance between entropic forces and thermal energy keeping the stress level at zero. At high pre-strain values (10–60%), heating results in significant contraction of the sample, i.e. an increase in the internal stress of the polymer strip. PDMS is a hyperelastic polymer where the stress is derived from the strain energy function. Combining such hyper elastic material with ultra-large strength 2D nanomaterials can enable a significant amount of stored strain energy that is released on photo-excitation.

We developed a new mathematical model for the thermal and photo-thermal actuations to predict the temperature profile of unstrained and strained actuators. In photo-actuation, the temperature profile in the sample can be expressed as a Gaussian distribution^14^:

6 $${\Delta }{T}_{n}(x,y)=c{e}^{-\frac{{(x-{\mu }_{x})}^{2}}{2{\sigma }_{x}^{2}}}\,{e}^{-\frac{{(y-{\mu }_{y})}^{2}}{2{\sigma }_{y}^{2}}}$$

where *µ* is the mean and *σ* the standard deviation. This leads to a relationship between purely thermal and photothermal actuations with the following equation (for more details on this model, see part 2 of the supplementary document):

7 $${\Delta }{T}_{n}(x,y)=\frac{-LW\,{e}^{-\frac{{(x-{\mu }_{x})}^{2}}{2{\sigma }_{x}^{2}}}\,{e}^{-\frac{{(y-{\mu }_{y})}^{2}}{2{\sigma }_{y}^{2}}}}{2\pi {\sigma }_{x}{\sigma }_{y}\,(erf(\frac{2{\mu }_{x}-L}{2\sqrt{2}{\sigma }_{x}})-erf(\frac{{\mu }_{x}}{\sqrt{2}{\sigma }_{x}}))(erf(\frac{2{\mu }_{y}-w}{2\sqrt{2}{\sigma }_{y}})-erf(\frac{{\mu }_{y}}{\sqrt{2}{\sigma }_{y}}))}\,{\Delta }{T}_{u}$$

where *ΔT*_*n*_ denotes the temperature change in photo-actuation and *ΔT*_*u*_ the temperature change in thermal actuation. It should be noted that the thermal actuation takes almost 20 minutes for uniform temperature equilibration in DMA compared to photo-actuation which takes about 5–10 s for stress to saturate.

To corroborate the model, both *wrinkle* and flat MoS_2_/PDMS nano composites were tested for heat-induced actuation in DMA TA-Q800 as presented in Figure S_11_. Obviously, both samples show similar thermal actuation, as in heat-induced actuation the polymer chains are responsible for induced stress, which is the same in both samples. From these results, a corresponding ΔT_n_ for each of photoinduced stress values of wrinkle and flat samples is obtained.

**Figure 10:**
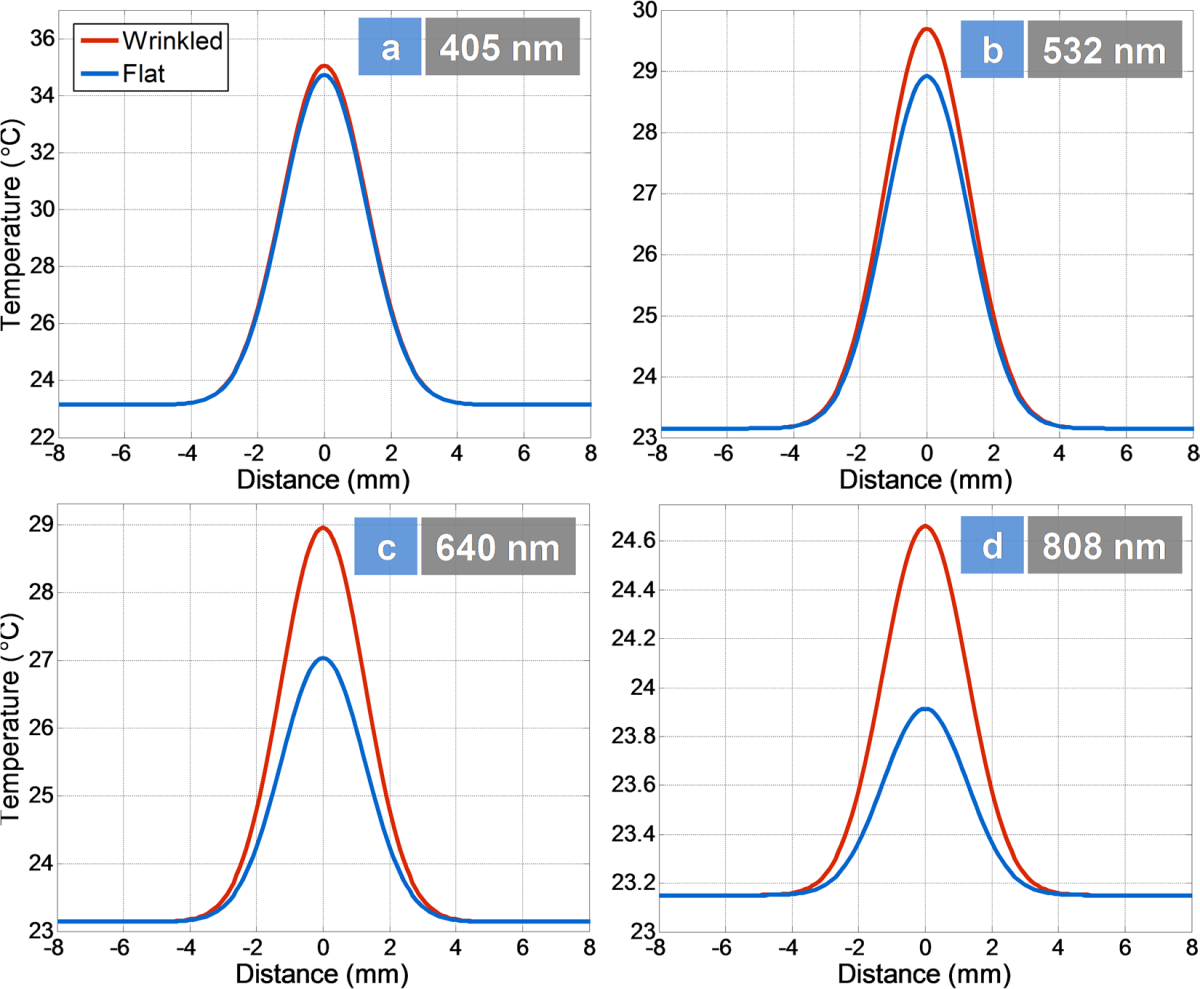
Simulated Temperature Profile: Gaussian temperature profile of wrinkle and unstrained actuators at different wavelengths: (**a**) 405 nm; (**b**) 532 nm; (**c**) 640 nm and (**d**) 808 nm.

Figure 10 presents the predicted temperature profiles based on our mathematical model for the unstrained and strained actuators at different wavelengths. The temperature of the sample as a function of the wavelength of the light for unstrained actuators was predicted as 35 °C (405 nm), 29 °C (532 nm), 27 °C (640 nm) and 23.9 °C (808 nm). The room temperature is 23 °C as a reference. For the strained actuators, the temperature of the sample as a function of the wavelength of the light is 35 °C (405 nm), 29.9 °C (532 nm), 29 °C (640 nm) and 24.6 °C (808 nm). At 405 nm both give rise to the same temperature profile according to Equation 7. However, between 532 nm to 808 nm, the locally strain engineered wrinkle samples gave rise to a higher temperature profile compared to the unstrained actuators. At the nanoscale, it is expected that the apex region of the wrinkle may enable higher temperature compared to flat surface. It is expected that thermal gradients exist between apex and base and heat is flowing from the apex to the base. The wrinkles have MoS_2_ layers partially directed with an angle to the surface of the polymer, which directs the generated heat of highly strained regions of the apex to the flat region, and to the polymer underneath.

Looking into the stress data further in Figure S_5_ (Suppl.), for instance, a photo-actuation of around 0.65 kPa for both flat and wrinkle actuators at 405 nm excitation wavelength will lead to a 2.2 °C temperature rise for ΔT_u_. This temperature rise is the uniformly distributed temperature, which is needed to generate an actuation of 0.65 kPa in heat-induced actuation. Using Equation 7 and the corresponding *µ* and *σ* values for these experiments, the equivalent temperature profile along the sample can be easily obtained. Table 1 lists the change in temperature values for wrinkle and flat samples at different excitation wavelengths at the center of samples (*x* = *y* = 0). The temperature profiles along the length of the samples are also depicted and compared for both wrinkle and flat photo-actuators. The wrinkled sample is predicted to give rise to a significantly increased temperature compared to their flat counterparts on photoactivation, resulting in improved mechanical response.

**Table 1:** Change in temperature in heat-induced and photo-induced actuations for unstrained and strained samples.

	Δs (kPa)		ΔT_u_ (°C)		ΔT_n_ (°C)	
	*wrinkled*	*flat*	*wrinkled*	*flat*	*wrinkled*	*flat*
405 nm	0.67	0.65	2.29	2.22	11.93	11.58
532 nm	0.3675	0.3239	1.26	1.11	6.55	5.76
640 nm	0.3259	0.2185	1.12	0.75	5.8	3.89
808 nm	0.0849	0.0429	0.29	0.15	1.51	0.76

## Discussion

In this paper, we present a new effect called the straintronic-photothermal effect, which is the intimate coupling of strains, band gaps, optical absorption and photothermal response in 2H-MoS_2_ hyperelastic nanocomposites. Compared to applying globalized strains with a mechanical gadget, localized nanoscale strain engineering through strain transfer from a polymer or other means is highly desirable. While change in band gaps has been used to modulate electronic properties of devices, our results are the first application of band gap effects and straintronic effects in MoS_2_ for mechanical actuation. As the bandgap of 2H-MoS_2_ is decreased due to localized strains, the optical absorption and the thermal and mechanical response is enhanced. The localized strains were estimated in the wrinkles accurately through an analytical strain model for van der Waals packed films based on the original work by Vella *et al*.^39^. The strains from 100 wrinkles showed anywhere between 0.2–1.3% strains. This model is useful for quasi-continuous/nanoparticle films based on liquid exfoliated TMDs. The photoluminescence study shows that the direct electron transition is still dominant in local strain engineered few layer additives. The shift in the A exciton resonance peak by 30 meV is clear evidence of the mechanism that the strength of the A-exciton resonance is responsible for the overall chromatic mechanical effect. Straining the layers also increased the PL intensity suggesting strains have a direct effect on the band structure in 2H-MoS_2_. A new model that relates the purely thermal effect versus Gaussian photomechanical effect suggests that photomechanical actuation in TMD based nanocomposites is a chromatic thermal effect coupled to the optical absorption of the semiconductor under strains. The straintronic-photothermal effect only occurs at thickness below 100 nm, and therefore access to nanoscale property is necessary to see this effect. Above 100 nm, bulk behavior dominates, and thus this effect vanishes completely, and strain engineering has no effect on subsequent photo-thermal response.

Combining 2D nanomaterials with hyperelastic materials provides new opportunities in photo-thermal transducers due to the high strain energy densities, high elastic modulus, and enhanced light matter interactions in these materials. We believe that 2D TMD based nanocomposites are one of the few materials to show intrinsic chromatic mechanical response as well as well as a straintronic photomechanical response with tunability of stress due to change in band gap effects, all of which can be exploited for light driven transducers on the macroscopic, millimeter and microscopic scales. In the past, graphene based hyperelastic nanocomposites have shown tunable photo-thermal response^43,44^. However, they do not exhibit chromatic mechanical response. Compared to that, TMDs are exciting materials as they possess an intrinsic band gap at 1.8–1.9 eV, which is tunable with strains. While the wrinkles are not periodic in our study and the strain distribution is non-uniform, making the wrinkles periodic using chemical vapor deposition (CVD) of TMDs with precise control of number of layers and strain transfer can enable more repeatable wrinkled architectures for tunable photo-thermal actuation. Further, the thermal gradients between the apex and base of the wrinkles enables heat transfer into the polymer. One can also tune the thermal and mechanical response to larger wavelengths through traditional plasmonic architectures by coating the wrinkles with 10–30 nm noble metals, resulting in exciton-plasmon interactions that can also tune optical absorption and mechanical response. This new straintronic-photothermic effect in TMD nanocomposites will be useful for the future development of microscopic transducers based on light, flexible straintronic devices, optoelectronic and photovoltaic devices/skins, substrates for surface enhanced Raman spectroscopy (SERS), chem-biosensing and energy harvesting.

## Material and Methods

### Sample Preparation

Few-layer MoS_2_ nanoflakes dispersed in ethanol solution was purchased from Graphene Supermarket. The lateral size of flakes was 100–400 nm, according to the manufacturer. Standard vacuum filtration process was used to deposit thin films of MoS_2_ nanoflakes with different thicknesses of 25–35 nm and 60–70 nm on mixed cellulose membranes with 25 nm pore size (EMD Millipore VSWP04700). The vacuum filtration process self-regulates the deposition rate of nanoflakes on the filter membrane to produce an evenly distributed thin film of MoS_2_. The thin film of MoS_2_ was subsequently transferred to strained and non-strained Polydimethylsiloxane (PDMS) substrate using an acetone bath that dissolved the overlaying filter membrane. PDMS silicone elastomer was obtained from Dow Corning (Sylgard 184). PDMS is a two part solvent-free flexible silicone organic polymer in the form of a base compound with a separate hydrosilane curing agent that acts as a cross-linker. The term cross-linking ratio refers to the ratio of PDMS cross-linker to the base compound. The PDMS base was mixed in 1:10 ratio to the cross-linker and then deposited on a glass slide. A standard spin coating process at 600 rpm for 30 seconds produces ~140 μm thick film of PDMS on the glass slides. The PDMS films are then cured at 120 °C for 20 minutes and post-cured for an additional 12 hours at room temperature before they were cut into 50 mm × 8 mm strips and installed in a hand-made setup for the transfer process under strain. After successfully transferring a thin and continuous film of MoS_2_ to the PDMS substrate, the strain is released, resulting in a wrinkled film of 2D material on top of elastomeric substrate.

### Microscopy Imaging

HRTEM was conducted using an FEI Titan transmission microscope operating at 300 kV at Brookhaven National Laboratory. The sample was prepared by dropping one drop of exfoliated MoS_2_ in ethanol solution on lacey-carbon Cu grids (Ted Pella Inc., CA). The Cu grids were dried in air to make sure there are sufficient isolated flakes to be observed. SEM images were obtained using a JEOL JSM-7000F instrument at 4 kV of power and under an ultra-high vacuum, 10^−5^ Pa. Secondary electron detector was utilized at 10 mm working distance to capture high-resolution images of MoS_2_ features at magnifications as high as 100,000X. The AFM images were taken using a NaioAFM of Nanosurf in intermittent mode with a cantilever resonance frequency of ~148 kHz.

### Raman and Photoluminescence Spectroscopy

The Raman measurements were performed by the excitation laser line of 532 nm using a Horiba XploRA Raman system in ambient air environment. The power of the excitation laser line was kept below 1 mW to avoid inducing any heating effects. The laser beam was focused onto the surface of the samples using a 100X objective lens. The photoluminescence measurements were conducted by a Horiba Multiline Raman Spectrometer LabRAM HR Evolution with a 100x objective lens, 600 gr/mm, and 1% ND filter with a 532 nm excitation laser and laser power of 0.2 mW to avoid any damages to the samples.

### Resonant Raman Scattering and Raman Maps

Raman analysis and mapping was performed using a Renishaw inVia Raman microscope system, equipped with a multi-channel high-resolution Si-CCD camera. The measurements were performed in backscattering geometry, with a 632 nm He-Ne laser as the excitation source, and an optical filter with an incident power of ~1 mW. Raman mapping was carried out in a point-by- point mode, i.e. a full Raman spectrum was collected from each position in the selected area. The Raman maps were collected using an Leica microscope objective (50 X), and the laser spot focused on the sample to a diameter of ~1 µm. While a motorized stage moved the sample under the laser beam, spectra were successively acquired from the defined region of interest. The maps, acquired with the 1 µm step, from regions of 20 µm × 20 µm, consisted of 441 (=21 × 21) pixels. A full Raman spectrum was collected at each pixel and the data acquisition time for a typical map was ~16 hours. Such maps were then analyzed, and the desired spectral maps were generated. First, the curve fit parameters method based on a theoretical curve fitting was simultaneously employed to all 441 Raman spectra, and a full set of spectra parameters (peak intensities, peak widths, etc.) was calculated. Then, specific maps were generated by plotting the specific fitting parameters or their combinations, such as peak intensity ratios.

### Stress and Bending Test Experiments

The stress test experiments were done using a self-made automatic photomechanical test assembly, as was reported earlier on^14^. The bending test samples consist of nanocomposites made of one 80-μm-thick PDMS layer and one 35-nm-MoS_2_ layer in wrinkled and flat structures. The nanocomposites were cut in 12 mm × 4 mm strip, and have been clamped in one end, as shown in Fig. 7. The clamping end was illuminated with different wavelengths of lasers with the same laser power of 30 mW. The displacement at the free end of the samples was measured using a high-speed MicroTrak II laser displacement sensor (LTC-025-02). A custom LabVIEW program was developed to control the test equipment and collect/monitor the experimental data acquisition. All experiments were conducted in a climate-controlled laboratory.

## Electronic supplementary material

Supplementary Information
