# Supplementary material for: The Coupled Straintronic-Photothermic Effect
Source: Sci Rep. 2018 Jan 8;8:64. doi: 10.1038/s41598-017-18411-w (PMC5758642; doi:10.1038/s41598-017-18411-w)
Supplement: Supplementary file 1 — Supplementary Information [file 41598_2017_18411_MOESM1_ESM.pdf]

SUPPLEMENTARY DOCUMENTATION

The Coupled Straintronic-Photothermic Effect

*Vahid Rahneshin[1], Dominika Ziolkowska[2,3], Arthur McClelland[4], Jaya Cromwell[1], Jacek B. Jasinski[2] and Balaji Panchapakesan[1]**

[1]Small Systems Laboratory

Department of Mechanical Engineering

Worcester Polytechnic Institute, Worcester, MA 01609

[2]Conn Center for Renewable Energy Research

University of Louisville, Louisville, KY 40292

[3]Faculty of Physics, University of Warsaw

Pasteura 5, 02-093 Warsaw, Poland

[4]Center for Nanoscale Systems

Harvard University

Cambridge, MA 02138

**bpanchapakesan@wpi.edu*

Table of Contents

1. Analytical strain model for van der Waals packed films of nanoparticles: 3

Figure S_1: Schematic of wrinkle geometry and an individual nanoparticle at an arbitrary location *x* with a radius of curvature κx 4

Figure S_2: AFM image of large area of wrinkled sample. Insert shows two nanowrinkles right next to each other; AFM height image of three different regions are presented. 5

Table S_1: Statistical information of height, width, strain and percentage strains from 100 different wrinkles for 35 nm film. 6

Table S_2: Statistical information of the length and number of wrinkles per area for 35 nm film. 7

Table S_3: Statistical information of height, width, strain and percentage strains from 100 different wrinkles for 70 nm film. 8

Table S_4: Statistical information of the length and number of wrinkles per area for 70 nm film**.** 9

Figure S_3: Raman Spectroscopy Map of Flat/Unstrained and Strained Samples: Raman profile maps from (a) flat and (b) wrinkled samples. The profiles sampling is indicated by white dashed lines in Fig. 5 of the manuscript. Resonant Raman scattering was measured using 633 nm excitation source. Each profile represents 21 spectra collected each micron. The changes of A_1g, E_1g +XA and “b” peak intensities between flat and wrinkled samples are easily observable. 10

Figure S_4: Photoluminescence spectroscopy of plain PDMS and MoS_2/PDMS 11

Figure S_5: Tunable straintronic photothermal actuation of unstrained and strained actuators between 405 nm-808 nm photon wavelengths 12

Figure S_6: Transmitted power by the unstrained and strained samples at 405 nm to 808 nm wavelengths. 13

Figure S_7: Straintronic photo-thermal displacement of bending actuators as a function of frequency between 5 Hz to 30 Hz. 14

Figure S_8: High angle scanning electron microscopy of wrinkles before and after applying an external strain suggesting stability of wrinkles. Scale bars show 1 μm. 15

Figure S_9: Stress versus temperature profile of plain PDMS using DMA. Three different regions namely contraction, zero stress and expansion are presented. 16

2. Photo- and thermal- actuation analogy: a mathematical model 17

Figure S_{10}: Stress versus temperature profile of plain PDMS using DMA. High strain region. 19

Figure S_{11}: Stress versus temperature profile of unstrained and strained MOS_2/PDMS samples using DMA. 20

References 21

1. Analytical strain model for van der Waals packed films of nanoparticles:

Wrinkling and delamination of thin films adhered to a polymer substrate was studied earlier by Vella et al [1]. The wrinkle profile for such films can be approximated by a cosine function in the form of the following equation [1]:

$$w = f(x) = \frac{\delta}{2}\left[1 + cos\frac{2\pi x}{\lambda}\right] \qquad (S_1)$$

where δ and λ are the height and width of the wrinkle. According to the classical theory of elasticity of plates developed by Lagrange, the stress and strain in an incremental element with an arbitrary small deflection *w(x, y)* can be stated as [2]:

$$\varepsilon_x = \frac{\partial u}{\partial x} \quad , \quad \varepsilon_y = \frac{\partial v}{\partial y} \qquad (S_2)$$

$$\sigma_x = \frac{-Ez}{(1-\nu^2)}\left(\frac{\partial^2 w}{\partial x^2} + \nu\frac{\partial^2 w}{\partial y^2}\right) \qquad (S_3)$$

$$\sigma_y = \frac{-Ez}{(1-\nu^2)}\left(\frac{\partial^2 w}{\partial y^2} + \nu\frac{\partial^2 w}{\partial x^2}\right) \qquad (S_4)$$

Where:

$$u = -z\frac{\partial w}{\partial x} \quad , \quad v = -z\frac{\partial w}{\partial y} \qquad (S_5)$$

with *z* the distance from the mid-plane of the plate. For a curved plate, the radius of curvature κ_x at an arbitrary point can be calculated based on the following equation:

$$\frac{1}{\kappa_x} = \frac{-\frac{\partial^2 w}{\partial x^2}}{\left\{1+(\frac{\partial w}{\partial x})^2\right\}^{3/2}} \qquad (S_6)$$

In composite films of nanoparticle, the radius of curvature of individual components, especially those inside the film, can be assumed equal to that of the film. As a result, based on the radius of curvature κ_x, the strain in each individual nanoparticle can be estimated as:

$$\varepsilon_x = \frac{z}{\kappa_x} \quad \text{(S7)}$$

Introducing Eq. S1 into Eqs. S6 and S7, the radius of curvature and strain in each individual nanoparticles at an arbitrary part of the wrinkle can be found as:

$$1/\kappa_x \cong -\frac{2\pi^2\delta}{\lambda^2} cos \frac{2\pi x}{\lambda} \quad \text{(S8)}$$

$$\varepsilon_x(x, z) \cong -\frac{2\pi^2 z\delta}{\lambda^2} cos \frac{2\pi x}{\lambda} \quad \text{(S9)}$$

with *x* being the horizontal distance from apex of the wrinkle and *z* the distance from the mid-plane of the individual nanoparticle (Figure S1).

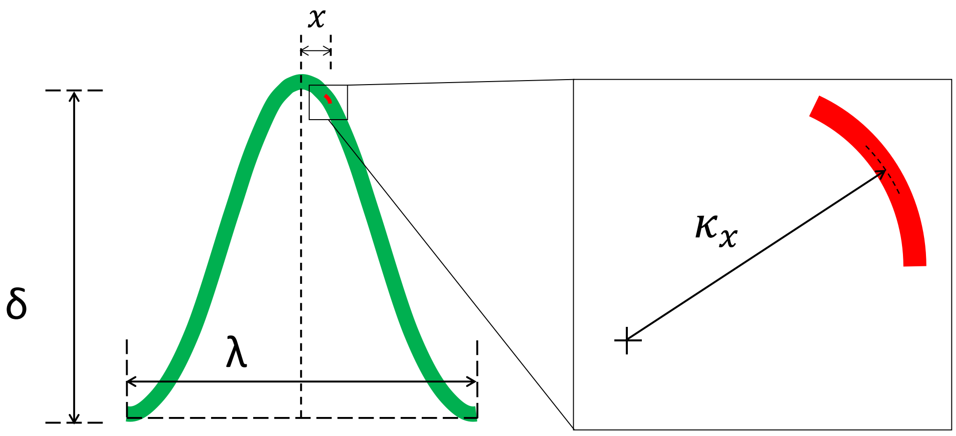

Figure S1: Schematic of wrinkle geometry and an individual nanoparticle at an arbitrary location *x* with a radius of curvature κ_x

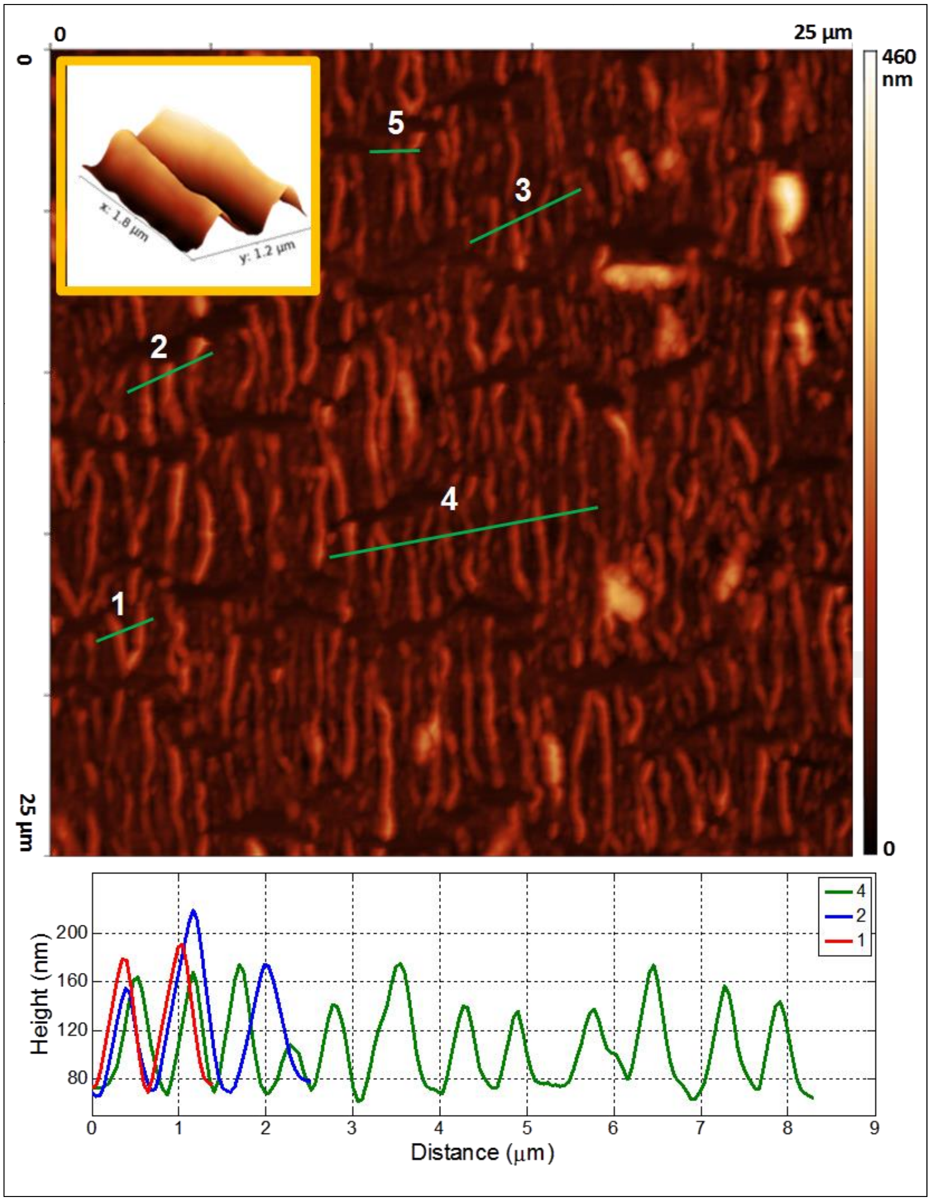

Figure S2: AFM image of large area of wrinkled sample. Insert shows two nanowrinkles right next to each other; AFM height image of three different regions are presented.

height, δ (nm)	135	113	131	168	105	82	147	97	131	115	108	121	82	123	100
width, λ (nm)	488	440	600	645	540	555	578	425	652	441	643	427	448	473	625
strain ε	0.0109	0.0112	0.0070	0.0078	0.0069	0.0051	0.0085	0.0103	0.0059	0.0114	0.0050	0.0128	0.0079	0.0106	0.0049
ε (%)	1.09	1.12	0.70	0.78	0.69	0.51	0.85	1.03	0.59	1.14	0.50	1.28	0.79	1.06	0.49
height, δ (nm)	113	105	97	74	65	106	220	147	55	183	38	27	61	118	73
width, λ (nm)	468	455	562	442	435	551	640	643	719	663	330	365	310	574	495
strain ε	0.0099	0.0098	0.0059	0.0073	0.0066	0.0067	0.0103	0.0068	0.0020	0.0080	0.0067	0.0039	0.0122	0.0069	0.0057
ε (%)	0.99	0.98	0.59	0.73	0.66	0.67	1.03	0.68	0.20	0.80	0.67	0.39	1.22	0.69	0.57
height, δ (nm)	132	76	35	95	83	58	81	125	96	74	104	95	53	37	53
width, λ (nm)	437	570	410	510	525	550	513	700	580	553	580	620	480	650	650
strain ε	0.0133	0.0045	0.0040	0.0070	0.0058	0.0037	0.0059	0.0049	0.0055	0.0047	0.0059	0.0048	0.0044	0.0017	0.0024
ε (%)	1.33	0.45	0.40	0.70	0.58	0.37	0.59	0.49	0.55	0.47	0.59	0.48	0.44	0.17	0.24
height, δ (nm)	68	86	96	51	57	157	34	98	127	92	118	14	73	177	59
width, λ (nm)	630	450	640	340	525	775	435	585	875	623	734	341	402	971	413
strain ε	0.0033	0.0082	0.0045	0.0085	0.0040	0.0050	0.0035	0.0055	0.0032	0.0046	0.0042	0.0023	0.0087	0.0036	0.0067
ε (%)	0.33	0.82	0.45	0.85	0.40	0.50	0.35	0.55	0.32	0.46	0.42	0.23	0.87	0.36	0.67
height, δ (nm)	58	129	82	102	31	14	157	79	78	69	64	118	95	115	106
width, λ (nm)	462	680	560	657	374	360	782	602	588	433	668	717	581	595	573
strain ε	0.0052	0.0054	0.0050	0.0045	0.0043	0.0021	0.0049	0.0042	0.0043	0.0071	0.0028	0.0044	0.0054	0.0063	0.0062
ε (%)	0.52	0.54	0.50	0.45	0.43	0.21	0.49	0.42	0.43	0.71	0.28	0.44	0.54	0.63	0.62
height, δ (nm)	49	98	61	34	104	44	85	99	98	97	97	76	93	56	31
width, λ (nm)	371	706	692	446	743	429	532	546	643	613	522	479	567	461	275
strain ε	0.0069	0.0038	0.0025	0.0033	0.0036	0.0046	0.0058	0.0064	0.0046	0.0050	0.0069	0.0064	0.0056	0.0051	0.0079
ε (%)	0.69	0.38	0.25	0.33	0.36	0.46	0.58	0.64	0.46	0.50	0.69	0.64	0.56	0.51	0.79
height, δ (nm)	69	72	123	134	96	89	78	68	109	113	157	120	94	81	22
width, λ (nm)	523	603	622	738	535	576	522	471	649	635	572	561	655	398	330
strain ε	0.0049	0.0038	0.0061	0.0047	0.0065	0.0052	0.0055	0.0059	0.0050	0.0054	0.0092	0.0073	0.0042	0.0098	0.0039
ε (%)	0.49	0.38	0.61	0.47	0.65	0.52	0.55	0.59	0.50	0.54	0.92	0.73	0.42	0.98	0.39

Average height, δ (nm)	87	(STD=37.6)
Average width, λ (nm)	553	(STD=123)
Average strain ε	0.0060	(STD=0.002)
Average ε (%)	0.60	(STD=0.24)

Table S1: Statistical information of height, width, strain and percentage strains from 100 different wrinkles for 35 nm film.

Length (μm)														
2.39	1.59	0.88	0.96	0.75	3.06	0.92	4.62	1.26	1.34	1.38	1.91	2.91	1.33	3.65
1.25	1.87	1.71	1.47	4.78	2.97	1.3	1.51	3.99	3.24	3.41	3.33	3.75	3.88	1.51
2.5	1.33	1.34	0.87	0.74	1.76	1.5	3.27	1.31	2.75	2.99	1.83	1.3	2.52	1.61
1.47	1.55	1.57	1.77	2.21	2.13	3.05	1.06	2.78	1.65	1.99	2.4	2.74	2.35	2.84
2.63	3.65	4.15	3.88	1.35	1.45	0.57	1.11	5.11	5.14	4.12	1.2	1.67	6.47	3.52
3.35	0.88	2.45	2.16	1.5	1.78	3.53	1.44	0.61	1.58	2.16	1.67	0.53	2.29	1.68
2.14	5.61	2.08	2.73	3.08	2.11	2.46	3.01	1.92	1.84	4.59	2.72	3.27	3.32	1.38
3.06	1.39	2.97	1.42	4.15	2.46	3.15	3.58	0.83	1.31	0.76	1.23	2.1	1.92	2.1
2.75	3.64	2.88	5.33	4.31	4.34	3.99	2.02	2.47	1.3	0.4	0.81	3.13	2.01	1.59
1.11	1.1	2.74	1.62	2.46	2.48	2.67	2.47	4.51	4.01	3	0.84	0.85	1.75	0.94
2.28	3.79	3.38	1.64	3.45	1.75	1.6	1.88	2.55	0.86	2.82	2.25	1.93	2.78	5.16
2.32	3.82	2.55	4.21	4.14	1.23	0.91	3.29	4.42	3.32	4.4	3.32	1.26	1.89	5.02
2.61	5.15	5.23	5.06	1.18	1.16	2.02	4.64	4.84	0.7	0.66	1.27	1.86	2.44	1.54
1.75	1.56	1.16	1.85	1.48	1.25	2.9	2.63	1.9	2.43	0.96	0.84	0.82	1.68	1.52
3.32	4.18	4.32	4.04	0.69	1.03	1.4	2.25	2.85	2.29	1.38	1.06	3.11	1.62	1.29
1.92	1.19	1.46	2.63	2.57	3.4	4.06	1.63							

Average length, L (μm)	**2.373**	(STD=1.224)
Average island lengths (μm)	**4.151**	(STD=1.967)
Number of wrinkles*	**247**	
Wrinkles per area (μm^{-2})	**0.395**	

* in an area of 25×25 μm^2

Table S2: Statistical information of the length and number of wrinkles per area for 35 nm film.

height, δ (nm)	480	460	510	350	260	270	360	490	310	550	500	650	270	600	290
width, λ (nm)	1700	1700	1700	1300	1200	1300	1500	1400	1100	1800	1700	2050	1500	1900	1400
strain ε	0.0032	0.0031	0.0034	0.0040	0.0035	0.0031	0.0031	0.0048	0.0049	0.0033	0.0033	0.0030	0.0023	0.0032	0.0028
ε (%)	0.32	0.31	0.34	0.40	0.35	0.31	0.31	0.48	0.49	0.33	0.33	0.30	0.23	0.32	0.28

height, δ (nm)	250	450	600	250	430	520	740	560	470	260	370	430	350	345	170
width, λ (nm)	1000	1700	2000	1050	1500	1550	2400	1450	1700	1050	1700	1550	1650	1500	1100
strain ε	0.0048	0.0030	0.0029	0.0044	0.0037	0.0042	0.0025	0.0051	0.0031	0.0045	0.0025	0.0034	0.0025	0.0030	0.0027
ε (%)	0.48	0.30	0.29	0.44	0.37	0.42	0.25	0.51	0.31	0.45	0.25	0.34	0.25	0.30	0.27

height, δ (nm)	430	560	370	260	390	340	600	345	480	580	460	310	390	330	540
width, λ (nm)	1250	1550	1550	1200	1500	1750	1900	1250	1600	1750	1850	1100	1550	1350	2100
strain ε	0.0053	0.0045	0.0030	0.0035	0.0033	0.0021	0.0032	0.0042	0.0036	0.0036	0.0026	0.0049	0.0031	0.0035	0.0024
ε (%)	0.53	0.45	0.30	0.35	0.33	0.21	0.32	0.42	0.36	0.36	0.26	0.49	0.31	0.35	0.24

height, δ (nm)	310	330	270	230	280	540	570	470	610	490	360	300	390	370	210
width, λ (nm)	1450	1700	1500	1450	1400	1850	2000	1900	1900	1800	1500	1300	1400	1400	1250
strain ε	0.0028	0.0022	0.0023	0.0021	0.0027	0.0030	0.0027	0.0025	0.0033	0.0029	0.0031	0.0034	0.0038	0.0036	0.0026
ε (%)	0.28	0.22	0.23	0.21	0.27	0.30	0.27	0.25	0.33	0.29	0.31	0.34	0.38	0.36	0.26

height, δ (nm)	205	140	540	255	330	510	410	380	270	560	470	205	290	270	340
width, λ (nm)	1100	1100	1900	1250	1300	2000	1850	1400	1450	1800	1650	950	1600	1450	1550
strain ε	0.0033	0.0022	0.0029	0.0031	0.0038	0.0025	0.0023	0.0037	0.0025	0.0033	0.0033	0.0044	0.0022	0.0025	0.0027
ε (%)	0.33	0.22	0.29	0.31	0.38	0.25	0.23	0.37	0.25	0.33	0.33	0.44	0.22	0.25	0.27

height, δ (nm)	500	265	270	450	440	220	405	330	380	345	305	440	430	515	500
width, λ (nm)	2150	1650	1450	1300	1600	1600	1600	1700	1750	1650	1200	1550	1700	2200	2000
strain ε	0.0021	0.0019	0.0025	0.0051	0.0033	0.0017	0.0030	0.0022	0.0024	0.0024	0.0041	0.0035	0.0029	0.0020	0.0024
ε (%)	0.21	0.19	0.25	0.51	0.33	0.17	0.30	0.22	0.24	0.24	0.41	0.35	0.29	0.20	0.24

height, δ (nm)	160	590	330	530	330	280	440	280	440	250					
width, λ (nm)	1300	2200	1600	1950	1350	1500	1900	1800	2200	1300					
strain ε	0.0018	0.0023	0.0025	0.0027	0.0035	0.0024	0.0023	0.0017	0.0017	0.0028					
ε (%)	0.18	0.23	0.25	0.27	0.35	0.24	0.23	0.17	0.17	0.28					

Average height, δ (nm)	**387.18**	(STD=124.2)
Average width, λ (nm)	**1582**	(STD=301.4)
Average strain ε	**0.0031**	(STD=8E-4)
Average ε (%)	**0.31**	(STD=0.083)

Table S3: Statistical information of height, width, strain and percentage strains from 100 different wrinkles for 70 nm film.

Length (µm)														
2.29	2.08	1.63	2.51	1.77	5.98	13.43	6.2	2.34	7.53	10.77	10.05	6.82	2.14	2.44
3.55	9.31	8.49	8.96	9	2.21	2.88	2.66	1.47	1.04	5.72	8.22	8.05	4.89	6.35
9.2	8.28	3.63	9.81	7.6	7.09	1.99	1.95	2.95	3.47	3.54	3.25	2.89	5.39	4.89
4.95	4.93	2.46	2.07	1.48	7.63	6.86	8.86	7.96	5.54	8.63	4.46	3.33	3.21	10.43
5.13	5.18	8.4	9.33	10.3	6.05	6.66	5.65	4.85	6.99	4.23	2.81	1.82	2.67	2.1
1.75	2.74	2.89	3.9	3.93	3.77	0.98	4.14	4.7	5.33	4.86	2.05	4.43	2.62	5.52
2.18	2.89	2.32	1.19	1.14	6.17	5.9	0.82	2.6	5.19	1.96	4.63	9.4	6.83	8.22
2.93	8.08	5.97	2.67	4.47	3.64	6.75	5.24	3.78	8.63	10.24	12.42	13.14	3.16	2.09
2.5	2.31	4.14	1.24	3.79	3.18	3.44	3.93	3.37	9.15	8.77	6.98	4.01	3.6	4.22
5.28	2.81	4.31	4.07	1.53	5.34	3.51	1.61	1.45	2.46	1.96	3.09	2.32	2.59	2.11
1.54	3.08	3.47	5.41	4.99	3.02	5.94								

Average length, L (µm)	**4.755**	(STD=2.770)
Average island lengths (µm)	**5.271**	(STD=2.784)
Number of wrinkles*	**204**	
Wrinkles per area (μm^{-2})	**0.082**	

* in an area of 50×50 μm^2

Table S4: Statistical information of the length and number of wrinkles per area for 70 nm film**.**

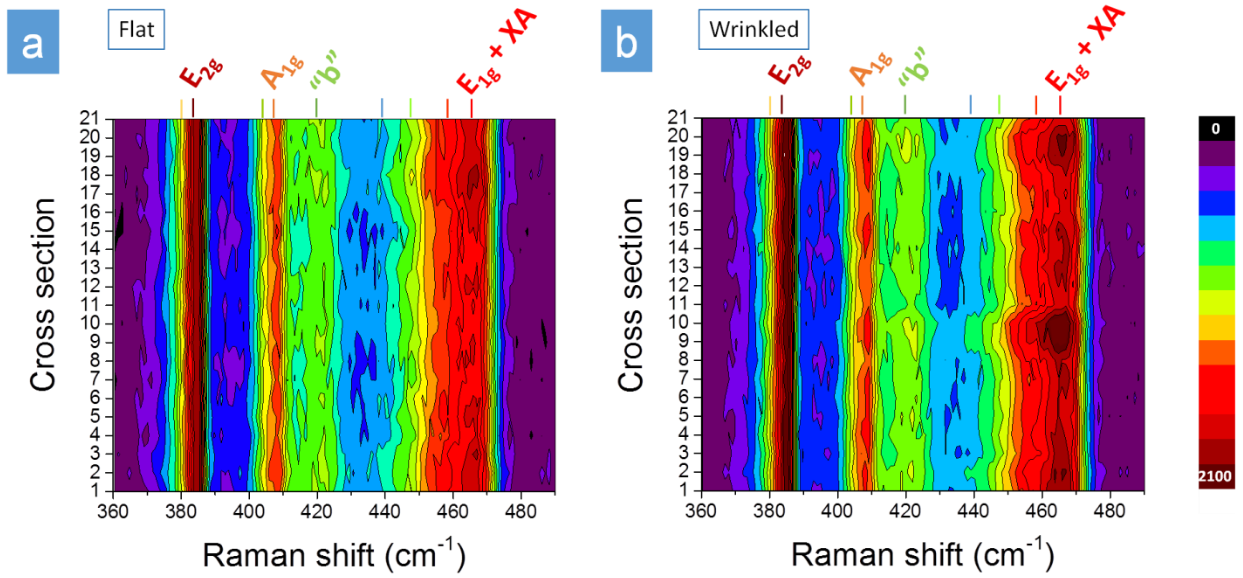

Figure S3: Raman Spectroscopy Map of Flat/Unstrained and Strained Samples: Raman profile maps from (a) flat and (b) wrinkled samples. The profiles sampling is indicated by white dashed lines in Fig. 5 of the manuscript. Resonant Raman scattering was measured using 633 nm excitation source. Each profile represents 21 spectra collected each micron. The changes of A_1g, E_1g +XA and "b" peak intensities between flat and wrinkled samples are easily observable.

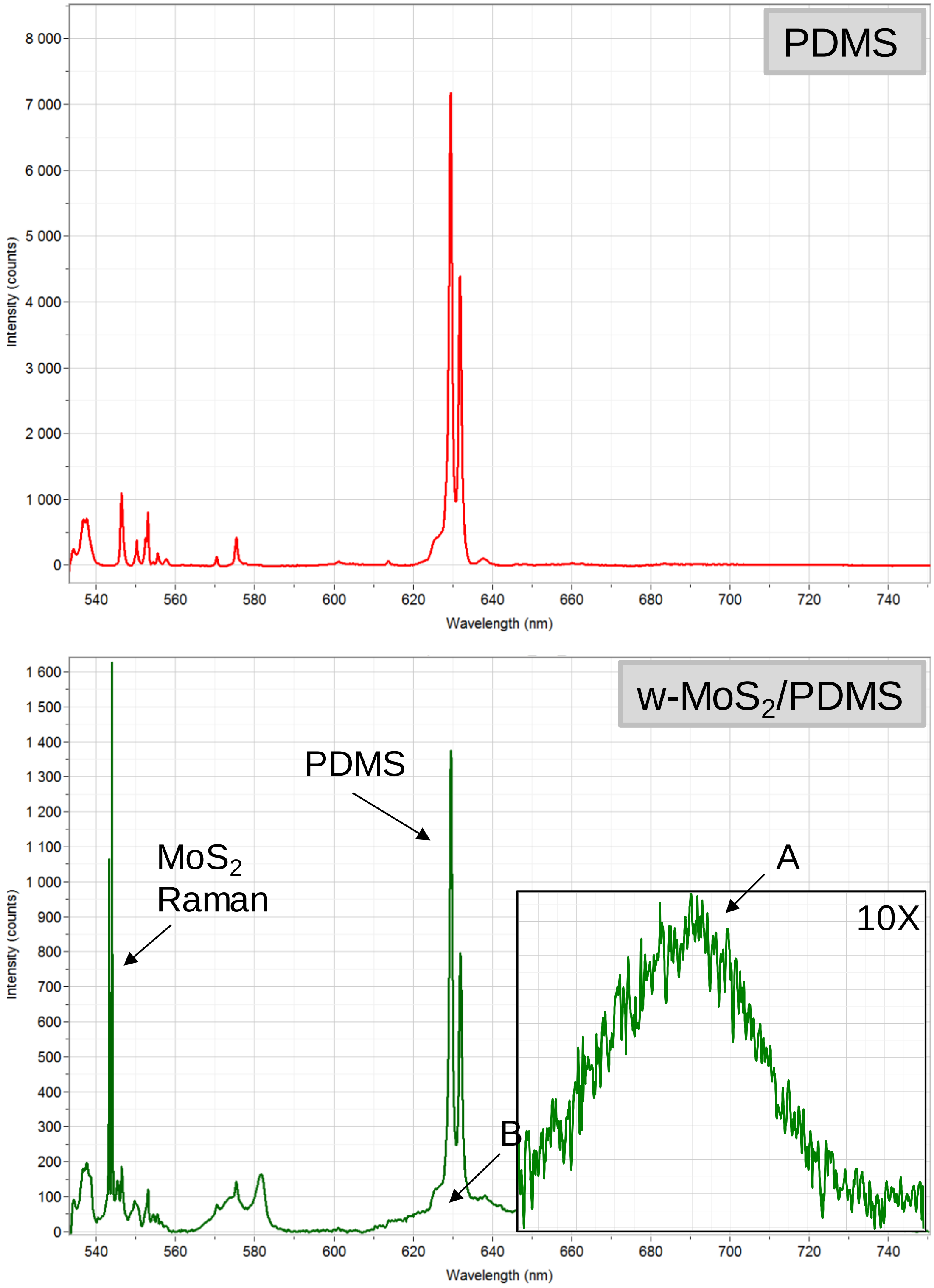

Figure S4: Photoluminescence spectroscopy of plain PDMS and MoS_2/PDMS

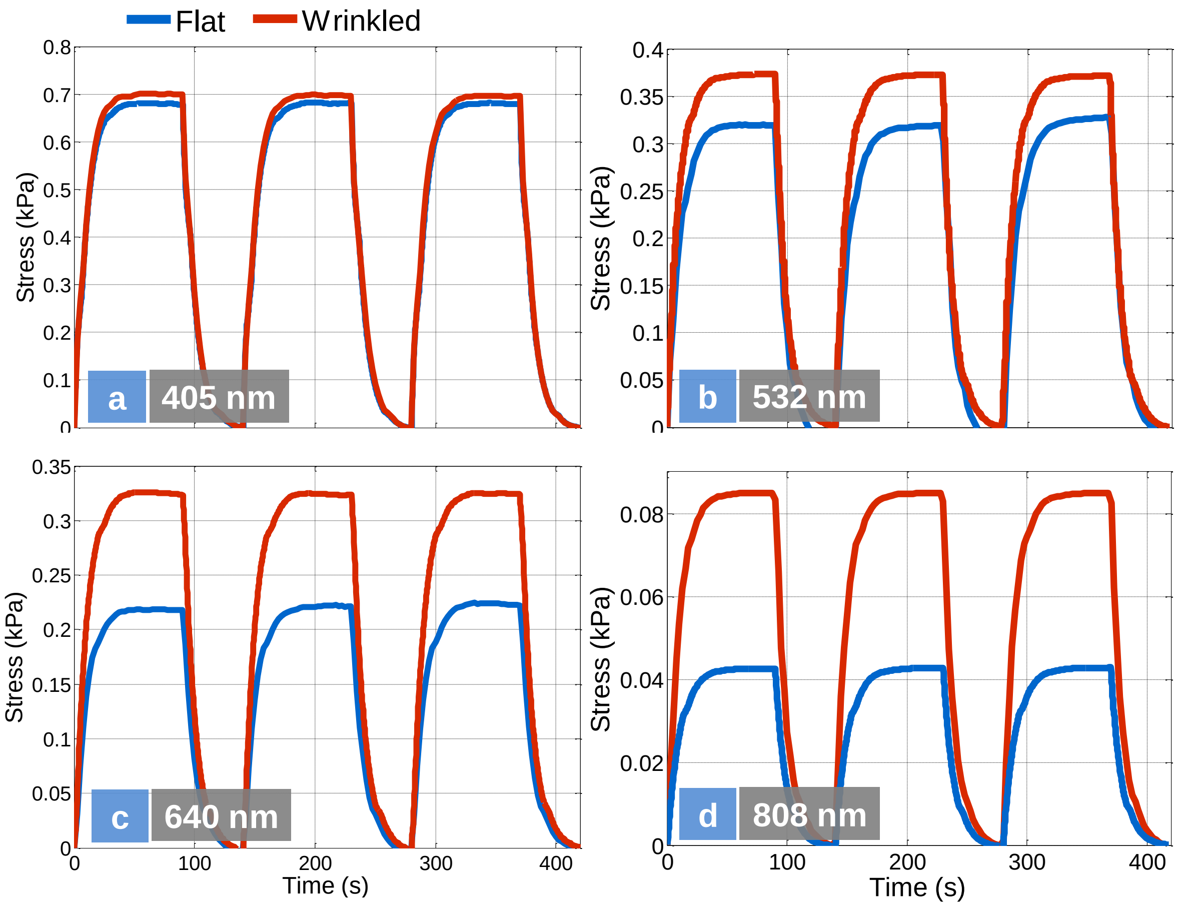

Figure S5: Tunable straintronic photothermal actuation of unstrained and strained actuators between 405 nm-808 nm photon wavelengths

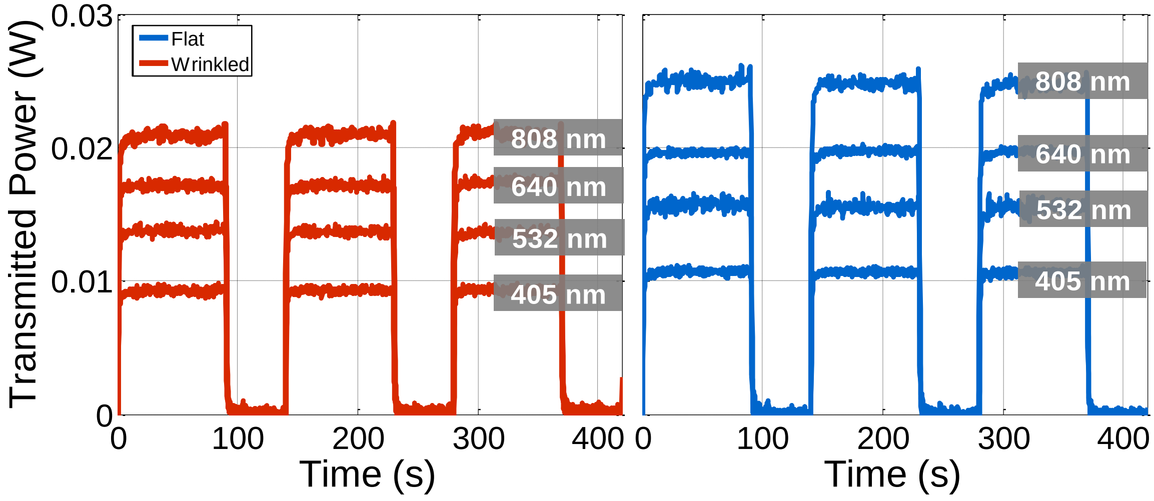

Figure S6: Transmitted power by the unstrained and strained samples at 405 nm to 808 nm wavelengths.

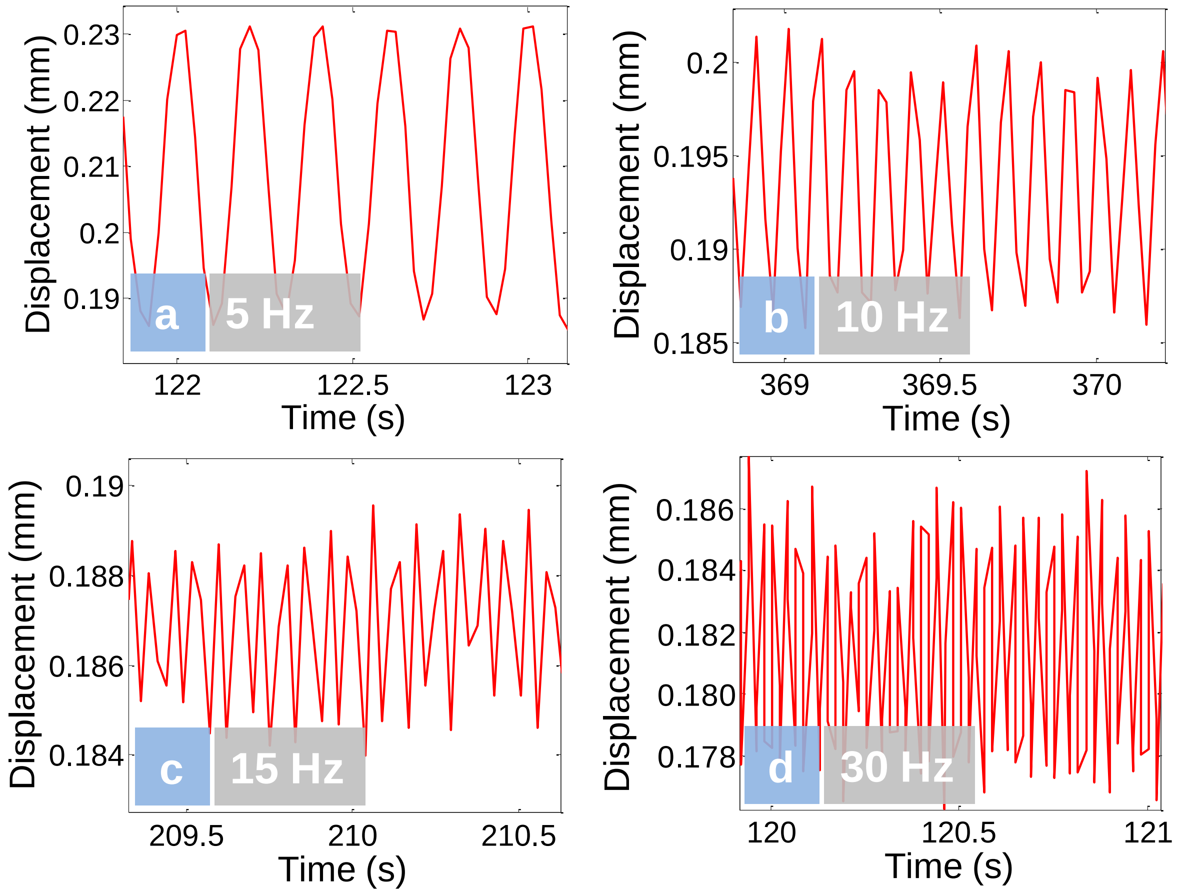

Figure S7: Straintronic photo-thermal displacement of bending actuators as a function of frequency between 5 Hz to 30 Hz.

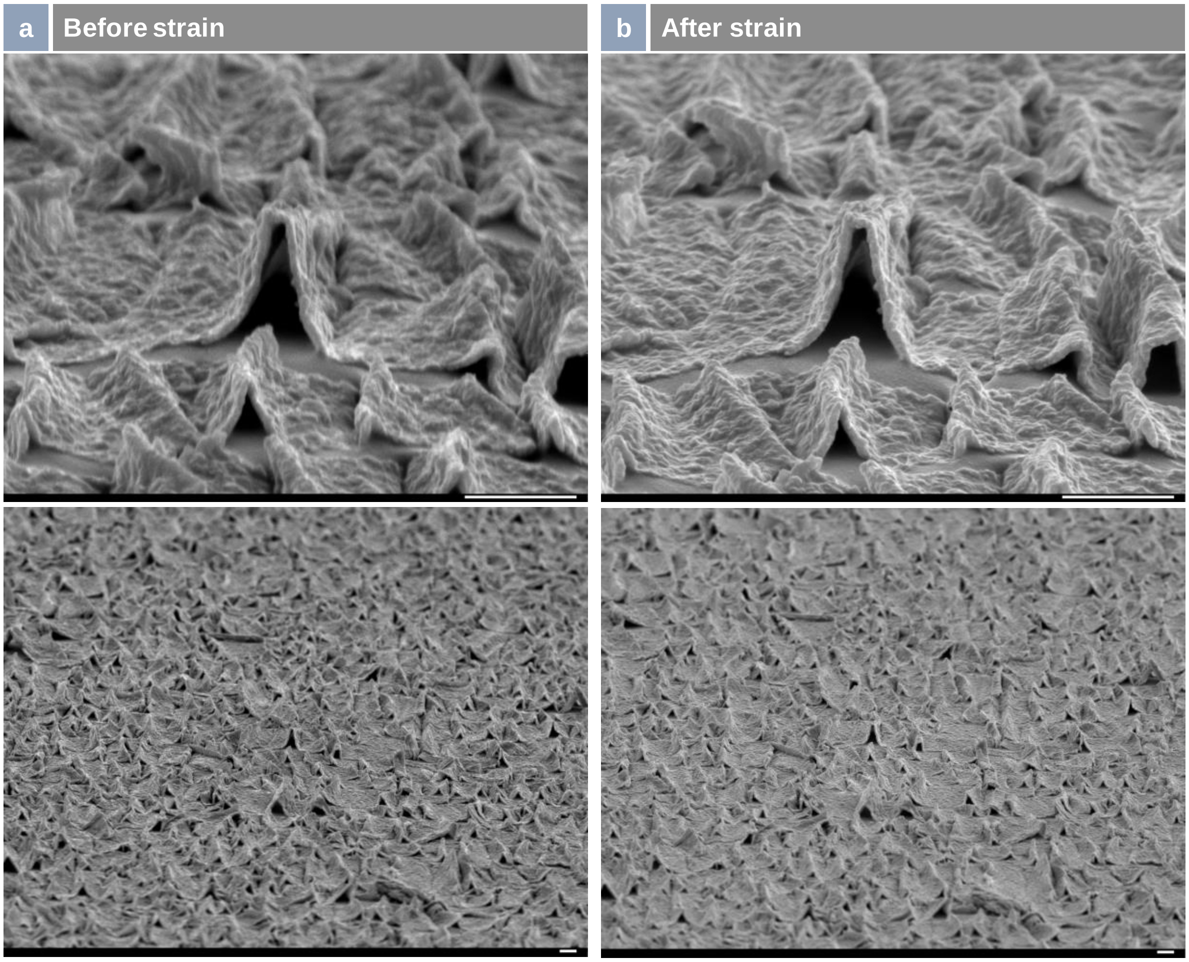

Figure S8: High angle scanning electron microscopy of wrinkles before and after applying an external strain suggesting stability of wrinkles. Scale bars show 1 µm.

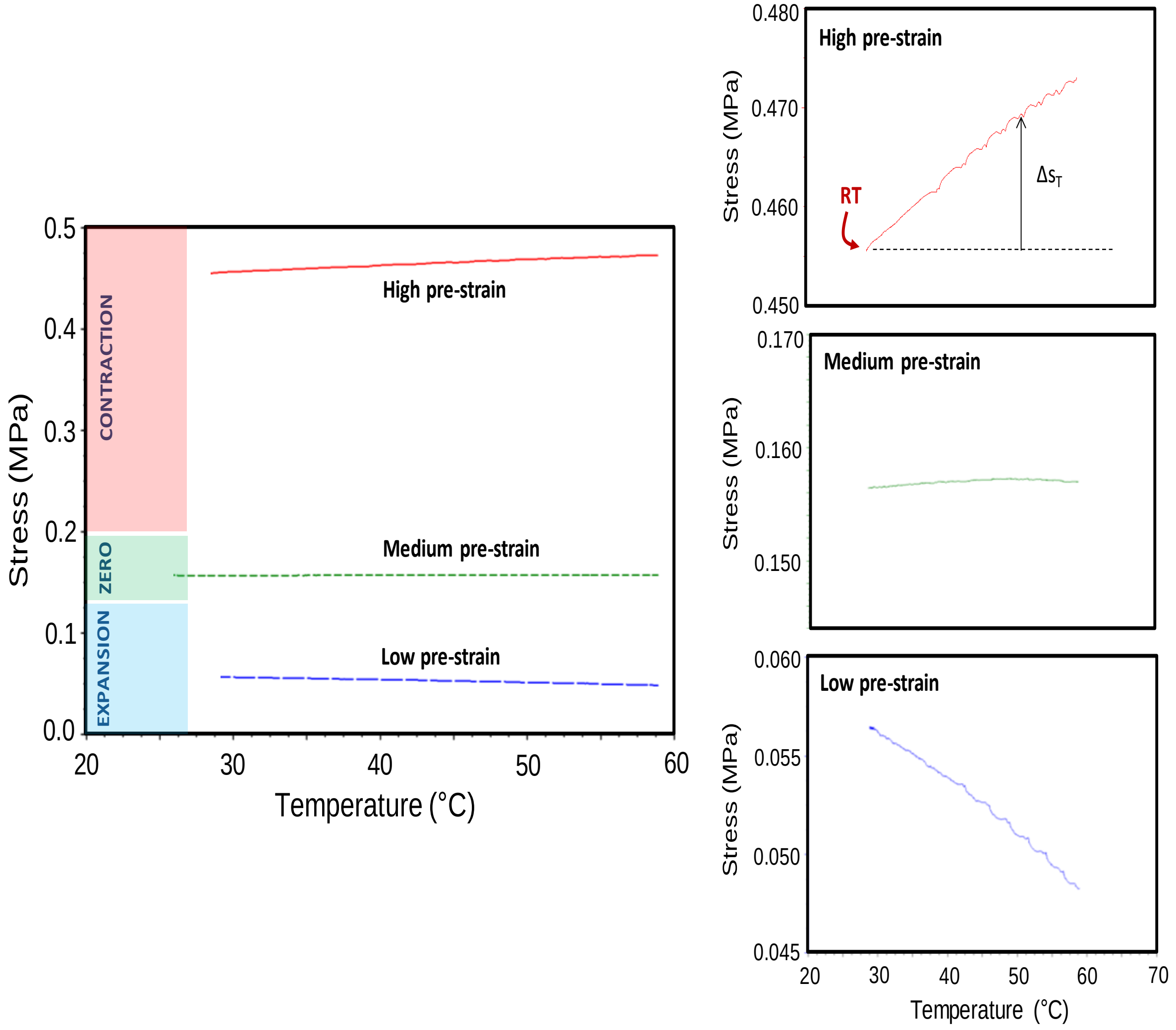

Figure S9: Stress versus temperature profile of plain PDMS using DMA. Three different regions namely contraction, zero stress and expansion are presented.

2. Photo- and thermal- actuation analogy: a mathematical model

In this part, a new mathematical model describing the relationship between purely thermal and photo-thermal actuations is presented. If we consider a specific actuation stress (Δs) resulted from photo-actuation of MoS_2/PDMS nanocomposites, and assuming the same amount of actuation Δs happens in pristine PDMS by heating up the sample, an equivalent temperature rise in the photo-actuation process is found by heat-induced temperature rise in DMA. Although the mechanism in both heat induced and photo induced actuation are purely thermal, the heat-induced stress resulted from thermal experiments in DMA is due to a uniform distribution of temperature (ΔT_u) around the polymer sample. As a result, the uniformly distributed temperature rise corresponded to this thermally induced Δs must be equalized to a Gaussian-distribution temperature profile (ΔT_n), which is the case for a pointed laser photo-actuation of MoS_2-based nanocomposites.

$$\Delta s^H = \Delta s^P = \Delta s \qquad (S_{10})$$

$$\Delta s \propto \Delta T_u \propto \Delta T_n \qquad (S_{11})$$

Where *H* denotes heat-induced actuation, *P* photo-induced actuation, *u* uniform distribution, and *n* Gaussian distribution. Supplementary Figure S_{10} shows a common experimental diagram for thermally induced actuation of pristine PDMS. The amount of heat transferred to the polymer chains because of temperature rise in the actuators is stated by the following equation:

$$Q = mC_p\Delta T \qquad (S_{12})$$

Where C_p is the specific heat capacity of the polymer. If we consider the steady state part of the thermal actuation and assume a uniform temperature profile along the thickness for thin samples, then we can dual integrate the heat profile along the length and width of the samples and find the relationship between ΔT_n in photo-actuation setup and heat-induced ΔT_u in DMA. Assuming, that the transduced Q for both

mechanisms is the same, the change in the temperature has an inverse relationship with the mass of the exposed part of the actuator. If we integrate the temperature profile along the exposed surface of the actuators in the two mechanisms, then:

$$m_u \Delta T_u = m_G \Delta T_n \quad (S_{13})$$

$$\int_{-w/2}^{w/2}\int_{-L/2}^{L/2} \rho_u t_u \Delta T_u \, dx\, dy = \int_{-w/2}^{w/2}\int_{-L/2}^{L/2} \rho_G t_G \Delta T_n \, dx\, dy \quad (S_{14})$$

Where *dx* and *dy* are the differential length and width elements, ρ the density, and *t* the thickness. From experimental data on MoS_2 based nanocomposites, the distribution of temperature change along the sample in photo-actuation is represented as a Gaussian distribution. The two-dimensional temperature relationship in photo-actuation is expressed by the following equation.

$$\Delta T_n(x,y) = c e^{-\frac{(x-\mu_x)^2}{2\sigma_x^2}} \; e^{-\frac{(y-\mu_y)^2}{2\sigma_y^2}} \quad (S_{15})$$

Where μ is the mean, σ the standard deviation and *c* a constant. In our photo-actuation system, μ is equal to zero for both *x* and *y* directions since the coordinate origin is placed at the center of the sample where the laser hits the nanocomposite. Experimental results for MoS_2/PDMS nanocomposites show that the square root of variance value ($2\sigma^2$) is approximately equal to the diameter of laser beam hitting the sample and is around 3.15, as reported in *Rahneshin et al.* [3]. Constant *c* is directly dependent on the value of photo-induced stress, which itself is a function of different parameters such as laser power, laser wavelength, the thickness of TMD layer, and geometrical pattern of the thin film. Substituting Eq. S_{15} into Eq. S_{14} and integrating over the volume of the sample yields the equivalent temperature relationships.

$$LW\Delta T_u = -2\pi c \sigma_x \sigma_y \left(erf\left(\frac{2\mu_x - L}{2\sqrt{2}\sigma_x}\right) - erf\left(\frac{\mu_x}{\sqrt{2}\sigma_x}\right)\right)\left(erf\left(\frac{2\mu_y - w}{2\sqrt{2}\sigma_y}\right) - erf\left(\frac{\mu_y}{\sqrt{2}\sigma_y}\right)\right) \quad (S_{16})$$

$$c = \frac{-LW}{2\pi\sigma_x\sigma_y \left(erf\left(\frac{2\mu_x - L}{2\sqrt{2}\sigma_x}\right) - erf\left(\frac{\mu_x}{\sqrt{2}\sigma_x}\right)\right)\left(erf\left(\frac{2\mu_y - w}{2\sqrt{2}\sigma_y}\right) - erf\left(\frac{\mu_y}{\sqrt{2}\sigma_y}\right)\right)} \Delta T_u \quad (S_{17})$$

Substituting Eq. S_{17} into Eq. S_{15} gives the equivalent temperature rise in the photo-actuation mechanism.

$$\Delta T_n(x,y) = \frac{-LW\, e^{-\frac{(x-\mu_x)^2}{2\sigma_x^2}}\, e^{-\frac{(y-\mu_y)^2}{2\sigma_y^2}}}{2\pi\sigma_x\sigma_y \left(erf\left(\frac{2\mu_x-L}{2\sqrt{2}\sigma_x}\right)-erf\left(\frac{\mu_x}{\sqrt{2}\sigma_x}\right)\right)\left(erf\left(\frac{2\mu_y-W}{2\sqrt{2}\sigma_y}\right)-erf\left(\frac{\mu_y}{\sqrt{2}\sigma_y}\right)\right)}\ \Delta T_u \qquad (S_{18})$$

This equation describes the relationship between purely thermal and photo thermal actuations.

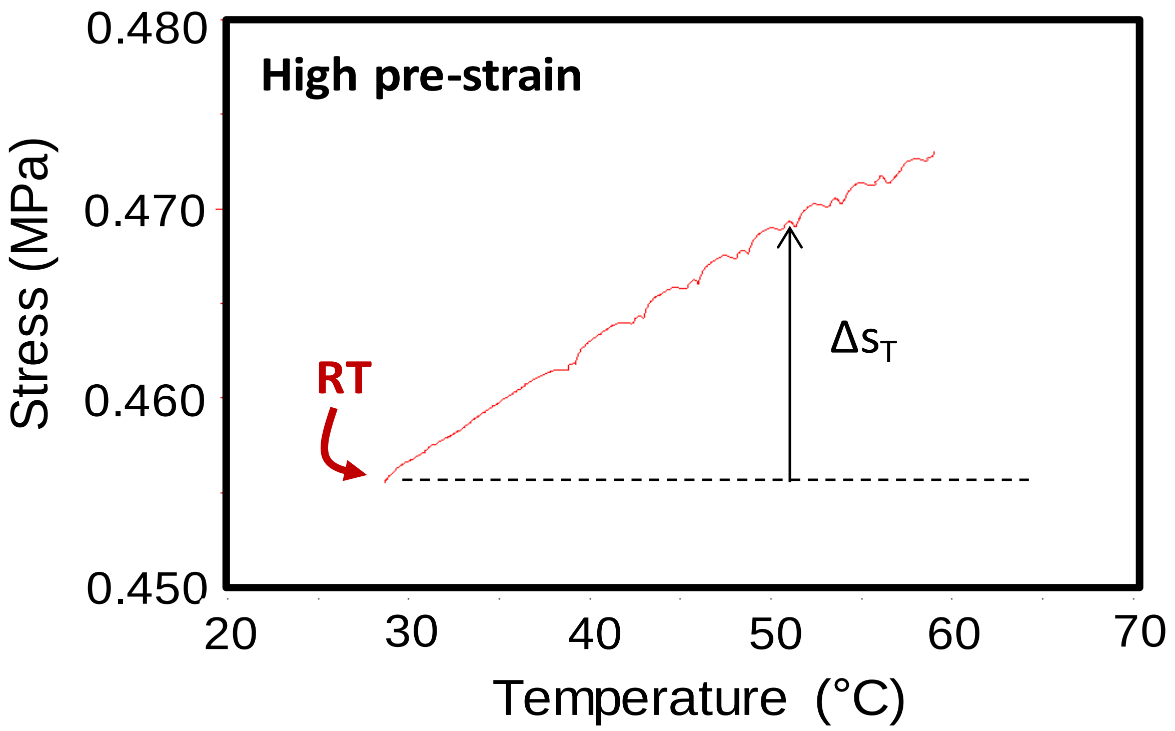

Figure S10: Stress versus temperature profile of plain PDMS using DMA. High strain region.

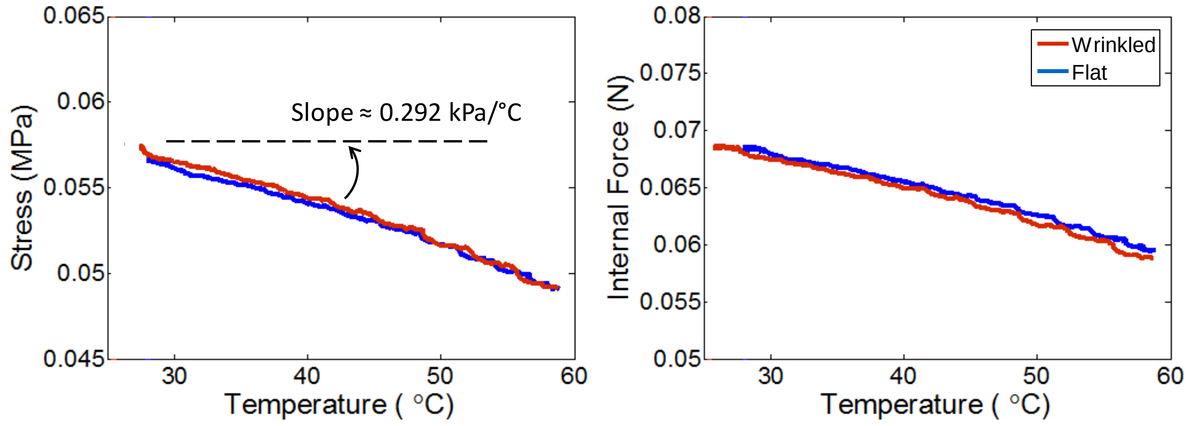

Figure S11: Stress versus temperature profile of unstrained and strained MOS_2/PDMS samples using DMA.

References:

1. Vella, D., et al., *The macroscopic delamination of thin films from elastic substrates.* Proceedings of the National Academy of Sciences, 2009. **106**(27): p. 10901-10906.
2. Mansfield, E.H., *The bending and stretching of plates*. 2005: Cambridge university press.
3. Rahneshin, V., et al., *Chromatic Mechanical Response in 2-D Layered Transition Metal Dichalcogenide (TMDs) based Nanocomposites.* Scientific Reports, 2016. **6**.
